# Automatised selection of load paths to construct reduced-order models in computational damage micromechanics: from dissipation-driven random selection to Bayesian optimization

**DOI:** 10.1007/s00466-016-1290-2

**Published:** 2016-04-09

**Authors:** Olivier Goury, David Amsallem, Stéphane Pierre Alain Bordas, Wing Kam Liu, Pierre Kerfriden

**Affiliations:** 1 School of Engineering, Cardiff University, Queen’s Buildings, The Parade, Cardiff, Wales CF24 3AA UK; 2 DEFROST Team, Inria Lille-Nord Europe, 40 avenue du Halley, 59000 Lille, France; 3 UMR-S 1159 Inserm – Université Paris VI Pierre et Marie Curie, 75005 Paris, France; 4 Department of Aeronautics and Astronautics, Stanford University, Mail Code 4035, Stanford, CA 94305 USA; 5 University of Luxembourg, Campus Kirchberg, G 007 Luxembourg, Luxembourg; 6 Department of Mechanical Engineering, Northwestern University, 2145 Sheridan Rd., Evanston, IL 60208 USA

**Keywords:** Model order reduction, Computational homogenisation, Reduced basis, Hyperreduction, Damage mechanics, Multiscale

## Abstract

In this paper, we present new reliable model order reduction strategies for computational micromechanics. The difficulties rely mainly upon the high dimensionality of the parameter space represented by any load path applied onto the representative volume element. We take special care of the challenge of selecting an exhaustive snapshot set. This is treated by first using a random sampling of energy dissipating load paths and then in a more advanced way using Bayesian optimization associated with an interlocked division of the parameter space. Results show that we can insure the selection of an exhaustive snapshot set from which a reliable reduced-order model can be built.

## Introduction

**Fig. 1:**
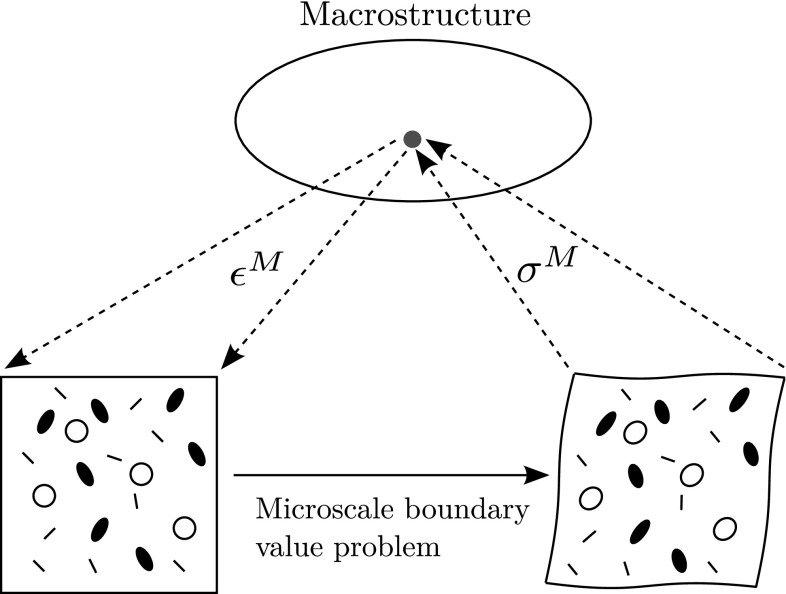
Semi-concurrent homogenisation procedure. At each macrostructural *quadrature point*, an RVE boundary value problem can be stated with boundary conditions dictated by the macrostrain at this *point*. Once the boundary value problem solved, the corresponding macrostress is evaluated as a spatial average of the microstress over the RVE

Multiscale modelling permits to take into account partial microscopic data when deriving engineering-scale working models. In solid mechanics, homogenisation is routinely used to obtain coarse-scale stress/strain relationships that are consistent with some statistical knowledge of the microstructure [1–4]. This is particularly useful when modelling complex phenomena that would require cumbersome heuristic inference if the subscale physics was ignored. In more advanced applications of upscaling concepts, the conservation laws of the coarse-scale medium themselves may be obtained from lower-scale data [5, 6]. Homogenisation can be seen as one particular class of upscaling technique, whereby coarse-scale models approximate the limit of the underlying microscale model when the scale ratio tends to zero [1, 7]. In the classical setting of micromechanics (see for instance [3, 4, 8]), the homogenisation process leads to two interlinked problems: a macroscale mechanical problem with homogeneous constitutive relations, and a microscale problem set over a representative volume element (RVE) of the microstructure, which is often interpreted as a material point of the homogeneous continuum. The solution to the macroscale problem defines a far-field loading for the RVE, usually in the form of boundary conditions. In turns, the solution of the RVE problem permits to find the homogenised coefficients of the coarse-scale constitutive relations, for instance by using micro/macro energy equivalence (Fig. 1).

RVE problems were traditionally solved approximately using analytical or semi-analytical approaches [2, 3, 9–11]. In the last 20 years, computational homogenisation has emerged as an interesting alternative approach [12–16], whereby the RVE problem is solved using direct numerical simulation. In linear elasticity, the homogenised constitutive relation can be pre-computed by performing a small set of material tests. The results of these tests are then assembled in the form of a homogenised Hooke tensor that can be readily used at the coarse-scale. In a nonlinear setting, a “naive” implementation of computational homogenisation requires to solve the RVE problem at every (quadrature) point of the macroscopic domain, which, although attractive due to its generality, may render the approach prohibitively expensive. A considerable amount of recent work aims at providing an answer to this dilemma. On the one hand, the community that relied heavily on semi-analytical approaches to solve RVE problems has developed methods to circumvent the limitations due to the restrictive assumptions upon which these approaches were traditionally based, at the cost of increased computational requirements. The (non-) uniform transformation analysis [17–19] (see also [20, 21]) and the Voronoi cell approach developed in [22] are remarkable instances of such developments. On the other hand, the community that relied primarily on computational homogenisation methods has tried to reduce the amount of RVE computations by using meta-modelling, often called meso-modelling in this context. Such developments include the R3M [23, 24] and the method developed in [25], which both rely on a combination of a proper orthogonal decomposition (POD) expansion [26, 27] for the solution field, and a surface response approach to interpolate the coefficients of this expansion over the space of admissible loading conditions. Our proposed approach is a further step in this direction, which bypasses the need for the surface response step and replaces it by reduced-order modelling (ROM).

Projection-based ROM is an increasingly popular technique for the fast solution of parametrised boundary-value problems. The key idea is to represent the parametric variations of the solution in a low-dimensional subspace. This subspace can be identified using the snapshot-POD [28–36], which compresses the posterior information contained in an exhaustive sampling of the parameter domain, or the reduced basis method [37–41], which searches for this attractive subspace in the form of a linear combination of samples chosen quasi-optimally via a greedy algorithm (“offline stage”). In a second stage, the boundary value problem is projected into this subspace, for instance by a Galerkin method, resulting in a reduced model of number of unknowns equal to the dimension of the attractive space. This reduced model is used to deliver an approximation of the solution to the parametric BVP for any set of parameters, and as such can be seen as an implicit interpolation method over the parameter domain (“online stage”). Early contributions concerning these type of methods have shown an increased accuracy compared to traditional response surface methods, for a given sampling of the parameter domain. Perhaps more importantly, these methods are based on approximation theories, and therefore “naturally” incorporate reliability estimates (e.g., [29, 35, 37, 40]).

In this paper, we propose to reformulate the nonlinear RVE problem as a parametrised boundary value problem, and subsequently to approximate it using projection-based ROM. Without loss of generality, we will consider an elastic damageable material represented by a network of damageable beams, with non-homogeneous material properties representing a random distribution of stiff inclusions into a softer matrix. The RVE problem will be parametrised by its far-field loading, represented by homogeneous Dirichlet conditions that belong to a vector space of dimension six (three in two 2D), the time evolution of the coefficients of the associated linear combination being a priori unknown, which effectively results in a parametric space of infinite dimension for the RVE. Therefore, our aim is to characterise the solution of the RVE problem for any history of the far-field load, within the restriction of ellipticity (which implicitly define the bounds of the parameter domain).

In a first attempt to approximate this parametrised solution, we will generate random loadings, enforcing a minimum amount of energy dissipation at each timestep and deploy the Galerkin-POD methodology to derive a reliable ROM. In a second, more advanced approach, we will develop a tailored reduced basis approach to sample the infinite-dimensional parameter space in a reliable and efficient manner. Our procedure relies on two major ingredients. Firstly, we will make use of a gradient algorithm to find points of the parameter space that need to be corrected during the iterates of the greedy algorithm. Although the gradient-based optimisation proposed in [39] is a promising strategy, we will make use of an alternative optimisation technique based on Bayesian optimisation [42]. More precisely, the load path of worst prediction will be found using a Gaussian process regression of an error indicator following [43]. The second ingredient of our approach is to coarsen the a priori infinite-dimensional parameter by applying the complex macroscopic load hierarchically, following a sequence of piecewise linear trajectories. Specifically, we will fully train a reduced basis method in a space of proportional loadings. We will then train a new reduced basis model in an enriched parameter domain, by representing the macroscopic load as a sequence of two piecewise linear loads, and further enrich our parameter domain in this hierarchical manner until a stagnation criterion is reached.

We will pay particular attention in the efficiency of the proposed strategy. In particular, projection-based ROM in the nonlinear setting is known to require an additional level of approximation to remain efficient, known as “hyperreduction” or “system approximation” [31–33, 38, 44–47]. We will make use of tailored version of the discrete empirical interpolation method (DEIM) [38, 46], which is, to date, the most widely used system approximation methodology. The original DEIM will be slightly modified to allow for the approximation of a vanishing nonlinear term in the balance equations of the discrete RVE problem. We will also propose a way to choose a good ratio between level of approximations in the truncation of attractive subspace versus system approximation.

The paper is organised as follows. In Sect. 2, we define the class of nonlinear homogenisation problems that we want to reduce, and explain how these problems can be parametrised. In Sect. 3, we develop specific model order reduction approaches based on the snapshot-POD and the reduced basis methodologies. We highlight the pros and cons of these two distinct approaches in the context of nonlinear homogenisation, and show results for each method. Conclusions are drawn in Sect. 4.

## Computational homogenisation setting

**Fig. 2:**
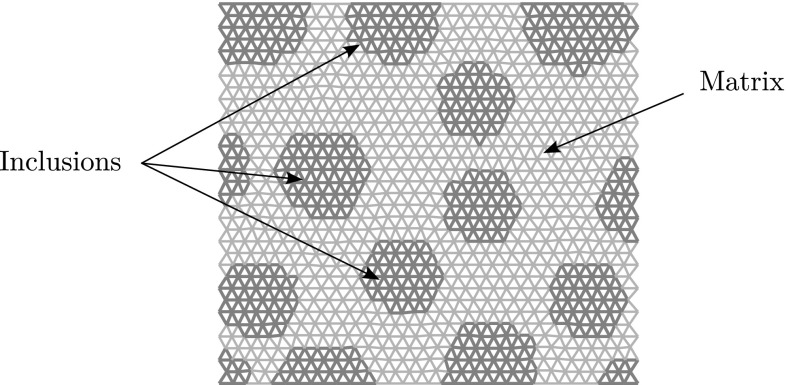
Lattice model of the computational representative volume element. Beams have different mechanical properties that depend on their location with respect to the distribution of heterogeneities in the computational domain. An arbitrary distribution of inclusions is chosen as a test case for this paper

**Fig. 3:**
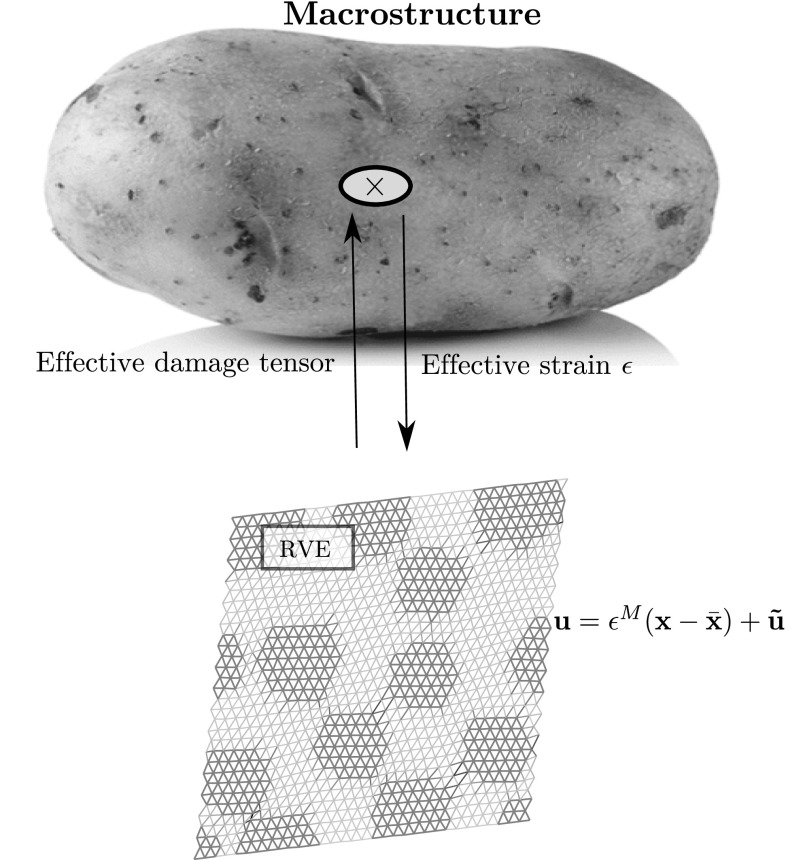
Schematic representation of computational homogenisation. The constitutive law of the macro-structure is defined implicitly. The macroscopic strain is applied as boundary condition to the RVE boundary value problem. In turn, the macroscopic stress field is extracted from the solution of the RVE problem using duality principles

We consider a generic RVE occupying domain $\Omega$ (Fig.  2), corresponding to a microscopically heterogeneous structure. The computational homogenisation approach that is considered in this work is a classical FE$^2$ scheme [12]: the RVE problem is to be solved numerically, under homogeneous Dirichlet boundary conditions, at every quadrature point of the macroscopic domain, which implicitly defines the nonlinear constitutive law at the macroscopic level. We will work under the assumption of small perturbations and isothermal mechanical evolution. The material studied in this paper is damageable elastic, but the methodology is general. In this paper, the RVE will be modelled by a 2D network of damageable beams (see for instance [48, 49] for more details), whose mechanical properties materialise heterogeneities (random distribution of stiff inclusions in our case). However, for the sake of simplicity, the idea of the approach will first be exposed in the context of continuum mechanics and then discretised, the formulation of the spatially discretised continuum-based or lattice-based model being similar (Fig. 3).

### RVE boundary value problem

At the RVE level, the displacement field is additively split into a fluctuation ${\tilde{\mathbf{u}}}$ and a smooth (or “macroscopic”) part ${\bar{\mathbf{u}}}{\text {:}}$

1 $$\begin{aligned} \mathbf{u}(\mathbf {x},\,t) = {\tilde{\mathbf{u}}}(\mathbf {x},\,t) + {\bar{\mathbf{u}}}(\mathbf {x},\,t), \end{aligned}$$

where the fluctuation ${\tilde{\mathbf{u}}}$ vanishes on the boundary $\partial \Omega$ of RVE domain $\Omega ,\,t$ denotes time, and the smooth part of the displacement belongs to a two-dimensional vector space,^1^

2 $$\begin{aligned} {\bar{\mathbf{u}}}(t) = \varvec{\epsilon }^M(t) ( \mathbf{x} - {\bar{\mathbf{x}}} ), \end{aligned}$$

where $\mathbf{x}$ is the position of a material point of the RVE, while ${\bar{\mathbf{x}}}$ is its centroid and $\varvec{\epsilon ^M}(t)$ gathers three scalar load coordinates that depend on the position of the corresponding material point of the macroscopic structure:

3 $$\begin{aligned} \varvec{\epsilon }^M(t) = \begin{pmatrix} \epsilon ^M_{xx}(t) &{} \epsilon ^M_{xy}(t) \\ \epsilon ^M_{xy}(t) &{} \epsilon ^M_{yy}(t) \end{pmatrix}. \end{aligned}$$

The mechanical equilibrium of the RVE is expressed by the principle of virtual work:

4 $$\begin{aligned} \int _\Omega \mathbf {\varvec{\sigma }^m}{\text {:}}\,\mathbf {\varvec{\epsilon }}(\mathbf {\varvec{\delta }u}) d\Omega = 0 , \quad \forall \mathbf {\varvec{\delta }u} \,\text {s.t.}\, \mathbf {\varvec{\delta }u}_{|\partial \Omega } = 0, \end{aligned}$$

where $\mathbf {\varvec{\sigma }^m}$ is the microscopic Cauchy stress, $\mathbf {\varvec{\epsilon }}$ is the strain operator that extracts the symmetric part of the gradient of a displacement vector, and ${\varvec{\delta u}}$ is a virtual fluctuation field.

The (damageable elastic) constitutive relation of the different micro-constituents of the material is assumed to be known at any time *t* of the analysis:

5 $$\begin{aligned} \mathbf {\varvec{\sigma }^m} = \mathbf {\varvec{\sigma }^m}\left( (\mathbf {\varvec{\epsilon }}(\mathbf{u}(\tau )))_{\tau \le t}\right) , \end{aligned}$$

where rate independence, causality and locality are assumed. The history dependence that appears in the previous expression is due to non-reversible damage processes such as plasticity or damage. For the sake of clarity, explicit history-dependance of the variables will be omitted in the remainder of the paper.

### Scale coupling

Following the classical computational homogenisation approach, the relationship between the macroscopic stress ${\varvec{\sigma }^M }$ and the macroscopic strain at time *t* and at an arbitrary macroscopic material point can be obtained by using the Hill–Mandel micro–macro energy consistency condition, which reads in the present context:

6 $$\begin{aligned} {\varvec{\sigma }^M }\left( \left( \varvec{\epsilon } \left( \mathbf {u}^{M}(\tau )\right) \right) _{\tau < t} \right) {\text {:}}\, {\varvec{\epsilon }^{M^\star } } = \frac{1}{|\Omega |} \int _{\partial \Omega } \left( {\varvec{\sigma }^m} \cdot \mathbf {n} \right) \cdot {\varvec{u}^\star } d\Gamma ,\nonumber \\ \end{aligned}$$

for any microscopic displacement ${\varvec{u}^\star }$ and any macroscopic strain ${\varvec{ \epsilon }^{M^\star } }$ related by the “strain averaging” Ansatz ${\varvec{u}^\star }(\mathbf {x}) = {\varvec{ \epsilon }^{M^\star } } ( \mathbf{x} - {\bar{\mathbf{x}}}).$ In the previous expression, $\mathbf {u}^{M}$ denotes the value of the macroscopic displacement field, and ${\varvec{\sigma }^m}$ is the microscopic stress field that is the solution of the RVE problem under far field load ${\bar{\mathbf{u}}}(\tau ) = \varvec{\epsilon } (\mathbf {u}^{M}(\tau )) ( \mathbf{x} - {\bar{\mathbf{x}}})$ for any time $\tau <t.$

Equation (6) leads directly to the definition of the macroscopic stress as a function of the macroscopic strain history $(\varvec{\epsilon } (\mathbf {u}^{M}(\tau )))_{\tau < t}{\text {:}}$

7 $$\begin{aligned} {\varvec{\sigma }^M }\left( \left( \varvec{\epsilon } \left( \mathbf {u}^{M}(\tau )\right) \right) _{\tau < t} \right) = \frac{1}{|\Omega |} \int _{\partial \Omega } \left( {\varvec{\sigma }^m} \cdot \mathbf {n} \right) \otimes ( \mathbf{x} - {\bar{\mathbf{x}}} ) d\Gamma ,\nonumber \\ \end{aligned}$$

which is subsequently used as constitutive equation for the macroscopic problem.

### Space discretisation and Newton solution algorithm

Equilibrium equation (4), after substitution of the microscopic constitutive relation, is discretised in space using for instance the finite element method (FEM):

8 $$\begin{aligned} \forall t , \, \forall \mathbf {\varvec{\delta }u} \, {\text {s.t.}} \, \mathbf {C} \mathbf {\varvec{\delta }u} = 0 , \quad {\varvec{\delta }}\mathbf {u}^T {\mathbf {f}}_{{\text {int}}}\left( (\mathbf {u}(\tau ))_{\tau \le t}\right) =0. \end{aligned}$$

This equation is complemented by the kinematic admissibility condition $\mathbf {u}(t) = {\tilde{\mathbf{u}}}(t) + {\bar{\mathbf{u}}}(t),$ where $\mathbf {u}$ denotes the vector of degrees of freedom of the FEM solution of the RVE problem at time *t*,  the vector ${\bar{\mathbf{u}}}(t)$ of degrees of freedom corresponding to the smooth “macroscopic” continuous field is known, and the vector of degrees of freedom ${\tilde{\mathbf{u}}}(t)$ corresponding to the continuous fluctuation field satisfies the discrete version of the vanishing boundary condition $\mathbf {C} {\tilde{\mathbf{u}}}(t) = 0.$

We will use a classical implicit time stepping procedure to discretise the RVE problem in time (i.e., integrate the history dependance in the microscopic constitutive relation). This will be further justified in the next paragraph. The continuous time interval ${\mathcal {T}}$ is discretised into $n_{t}$ subintervals $([t_{n} t_{n+1}]).$ Equilibrium and kinematic relations are enforced at successive discrete times $t_{n},$ while the continuous history dependency appearing in the constitutive relation is replaced by its discrete counterpart. The fully discrete, non-linear version of the system of Eq. (8) arising at time $t_\text {n}$ is solved using a Newton–Raphson (NR) algorithm. At each iteration of this algorithm, the following linearisation is computed and solved:

9 $$\begin{aligned} \forall \mathbf {\varvec{\delta }u} \, {\text {s.t.}} \, \mathbf {C} \mathbf {\varvec{\delta }u} = 0, \quad \mathbf {\varvec{\delta }u}^T\left( \mathbf {K}^i {\varvec{\Delta }}{\tilde{\mathbf {u}}}^{i+1} + \mathbf {r}^i \right) = 0, \end{aligned}$$

where $\mathbf {K}^i = \frac{\partial {\mathbf {f}}_{{\text {int}}}}{\partial \mathbf {u}}_{|\mathbf {u}^i}$ is the tangent stiffness matrix, $\mathbf {r}^i = {\mathbf {f}}_{{\text {int}}}({\tilde{\mathbf {u}}}^i+{\bar{\mathbf{u}}})$ is the residual vector and ${\varvec{\Delta }}{\tilde{\mathbf {u}}}^{i+1} = {\tilde{\mathbf {u}}}^{i+1} - {\tilde{\mathbf {u}}}^i = \mathbf {u}^{i+1} - \mathbf {u}^i$ (the second equality is only true if the smooth field is used as an initialisation for the NR algorithm, i.e., $\mathbf {u}^0 = {\bar{\mathbf{u}}}$) is the variation in the fluctuation vector.

**Fig. 4:**
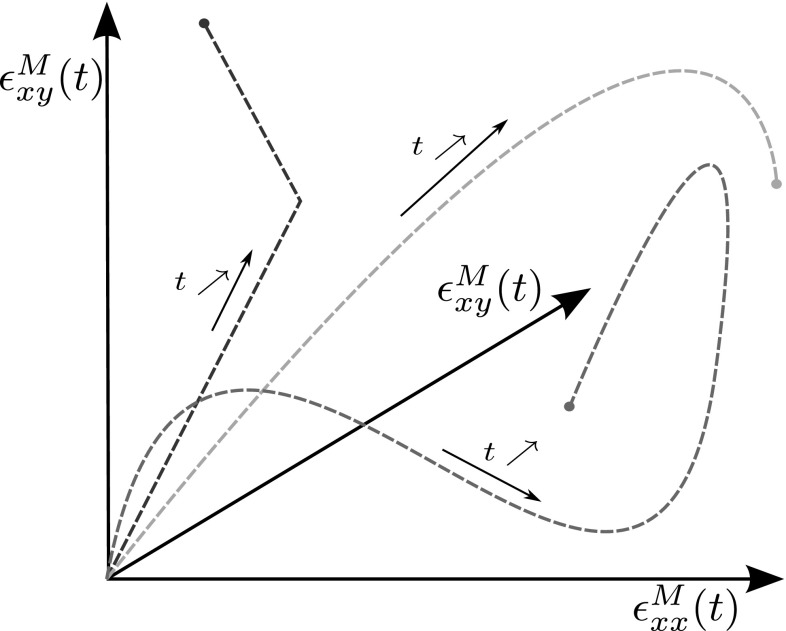
Representation of the parameter domain for the nonlinear RVE problem

### Parametrised RVE problem: description of the macroscopic load

In a FE$^2$ setting, the RVE problem is solved independently for every quadrature point of the macroscopic mesh. In order to apply our ROM technique, we recast the RVE problem as a family of boundary value problems subject to parameter dependency.

The parameters are the three independent components of the far field load tensor $\varvec{\epsilon }^M$ ($\epsilon ^M_{xx},\,\epsilon ^M_{yy}$ and $\epsilon ^M_{xy}$). Physically, they correspond to scalar descriptors of the loading history applied to the macroscopic material point. We emphasise the fact that these parameters are three functions of time, which is not a classical setting for model order reduction. This high (theoretically infinite) dimensionality is a challenge. Some realisations of the loading functions are depicted in Fig. 4.

The next step is to define the parameter domain, or in other words the space in which the three load functions can vary freely. This seems to be a largely problem-dependent issue, and we will focus the discussion on the class of rate-independent, damageable elastic materials. In this case, the first remark is that homogenisation loses its meaning once ellipticity is lost at the macroscopic level. Therefore, bounds are implicitly and collectively defined on the values of the loading functions by enforcing that the macroscopic tangent should remain positive definite. A second remark is that the speed at which the load is applied has no influence on the RVE solution; only the load path matters, which eliminates the need to describe loads that would be applied at different speeds but would essentially result in the same path.

We finally define a time integration scheme for the load history by forcing the macroscopic load to vary by a given amount between two successive time steps. More precisely,10Load parameter $\Delta l$ should be sufficiently small for the constitutive equations of the RVE to be correctly integrated and for the nonlinear solutions algorithms to converge.

Note that in this time-discrete setting, the number of parameters is two^2^ times the number of pseudo-time steps $n_t,$ which highlights the high-dimensionality of the problem.

## Reduction of the RVE boundary value problem

Our goal is to solve the balance equations of the RVE problem for any history of the macroscopic strain at reduced costs whilst retaining the accuracy of the computed macroscopic stress field. In order to do so, we postulate that for any load applied to the RVE, the fluctuation part of the displacement field can be approximated with an acceptable level of accuracy in a vector space of small dimension, called reduced space. This space being identified, we will find an approximation of the displacement field by looking for the amplitude (i.e., generalised coordinates) associated to the (few) basis vectors of this space. In this context, three questions arise:

- How can we identify the reduced space?
- How can we find the generalised coordinates in an efficient and stable manner?
- How can we evaluate the reliability of the approach?

The answer to the second question is now relatively well established in the literature. We will make use of a Petrov–Galerkin projection of the discrete set of balance equations (8) into the reduced space. More precisely, we will proceed in two stages: a first “ideal” Galerkin projection,^3^ followed by a second stage of approximation, called “system approximation” [32] or “hyperreduction” [45] to make the solution of the projected system computationally tractable.

The answers to the first and third questions are strongly intertwined, and we describe in the following paragraphs two different manners to approach the problem.

A POD-based approach looks for the best reduced space, in the sense of the minimisation of the projection error on average over the parameter domain. In practice, this optimization problem is reduced to a problem of minimum projection error over a representative set of solutions to the parametrised problem, the so-called snapshots [26]. In the case of large parametric dimensions, the sampling of the parameter domain needs to be done in such a way that it overcomes the “curse of dimensionality”, for instance by using quasi-random sampling techniques. The reliability of the approach can then be evaluated by resampling (cross-validation, bootstrap,$\ldots$) or other statistical tools. This approach suffers from two major drawbacks. Firstly, the optimality of the reduced space is established in an average sense over the parameter domain, which potentially results in inaccurate representation of outliers even for large dimensions of the reduced model. Secondly, the exhaustive sampling of the parameter domain might be prohibitively expensive, and is, in any case, inefficient if performed in a (statistically) uniform manner. The interested reader can find possible ways to tackle this difficult in [50]. Nonetheless, the POD-based methodology remains attractive because the optimization problem associated with the search of the reduced space can be solved using standard linear algebra tools, namely singular value decomposition.

The reduced basis [37] methodology aims at minimising the maximum projection error over the parameter domain. In practice, this is performed in a suboptimal manner using a greedy algorithm: the ROM is constructed iteratively by enriching the reduced space in order to decrease the error at the point of the parameter domain where some measure of projection error is at its largest. When reliable error estimates are available for the projection, the search for the highest level of error over the parameter domain is very efficient, which makes the approach very attractive. The sampling of the parameter domain is performed in a rational manner, which ensures that the construction of the ROM remains affordable. When error estimates are not available, the approach remains attractive in the context of large parametric dimensions. Indeed, the point of the parameter domain that corresponds to the largest level of projection error can be found using gradient-based optimization, whose numerical complexity may be made independent of the parametric dimension by using the adjoint methodology [39] to compute the sensitivities. In this setting, the “curse” of dimensionality can be overcome whilst retaining reliability of the ROM over the entire parameter domain.^4^

In the remainder of this section, we explore these two different possibilities for the reduction of the nonlinear RVE problem. We first propose a snapshot-POD approach, where the sampling is performed randomly, enforcing the random samples to undergo a minimum dissipation at each time step. In a second stage, we will develop a reduced basis approach for general loading, which allows for a more continuous approach which takes into account the error of the reduced model not only at the snapshots, but also between the snapshots thanks to a Gaussian process regression. We will propose specific ideas to overcome the “curse of dimensionality”.

### Galerkin projection of the governing equations in a reduced space

**Fig. 5:**
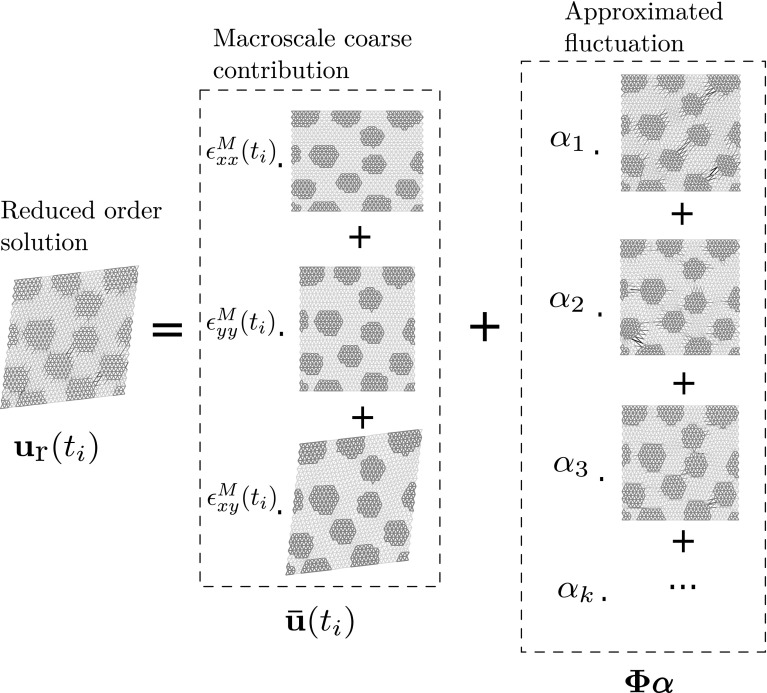
Surrogate model for the displacement field in the RVE. The surrogate is the sum of a macroscopic contribution (known a priori) and a fluctuation that is represented as a linear combination of basis vectors and obtained through the ROM

The fluctuating part of the displacement over the RVE^5^ is searched for in a reduced space ${\mathcal {U}}_{{\text {MOR}}} = {\text {span}}(({\varvec{\phi }}_i)_{i=1,N})$ of dimension *N* (see Fig. 5). The displacement is parametrised by the history of the far field load $( \mathbf {\varvec{\epsilon }^M}(t))_{t \in [0,T]},$ which will subsequently be denoted by $\mathbf {\varvec{\epsilon }^M}$ for simplicity. Mathematically, the surrogate for the displacement can be expressed at any time *t* as:

11 $$\begin{aligned}&\mathbf {u}\left( t;\,{\varvec{\epsilon }}^\mathbf{M}\right) = {\bar{\mathbf {u}}}\left( t;\,{\varvec{\epsilon }}^\mathbf {M}\right) + {\tilde{\mathbf {u}}}\left( t;\,{\varvec{\epsilon }}^\mathbf {M}\right) \approx {\bar{\mathbf {u}}}\left( t;\,{\varvec{\epsilon }}^\mathbf {M}\right) \nonumber \\&\quad + \sum _{i=1}^N {\varvec{\phi }_i} \alpha _i\left( t;\,{\varvec{\epsilon }}^\mathbf {M}\right) = {\bar{\mathbf {u}}}\left( t;\,{\varvec{\epsilon }}^\mathbf {M}\right) + {\varvec{\Phi }} {\varvec{\alpha }}\left( t;\,{\varvec{\epsilon }}^\mathbf {M}\right) . \end{aligned}$$

The degrees of freedom of the surrogate are the components of the vector of generalised coordinates ${\varvec{\alpha }}.$ In the previous equation, operator ${\varvec{\Phi }}$ is the matrix whose columns are the basis vectors of the reduced space ${\mathcal {U}}_{{\text {MOR}}}.$ In the following, for the sake of being more general, we will refer to the parameter as $\mu$ rather than being an explicit loading path defined by a macro-strain ${\varvec{\epsilon }}^{\mathbf {M}}.$

Substituting the trial and test vectors of balanced equation (8) by surrogate (11) leads to the Galerkin formulation

12 $$\begin{aligned} \forall t,\, \forall {\varvec{\delta \alpha }}, \quad&{\varvec{\delta \alpha }}^T {\varvec{\Phi }}^T {\mathbf {f}}_{{\text {int}}}\left( {\bar{\mathbf {u}}}\left( t;\,\mathbf {\varvec{\epsilon }^M}\right) + {\varvec{\Phi }} {\varvec{\alpha }}\left( t;\,\mathbf {\varvec{\epsilon }^M}\right) \right) = 0. \end{aligned}$$

This reduced nonlinear system of equations can be solved using a NR algorithm. At iteration *i* of this algorithm, we solve the linear system

13 $$\begin{aligned} {\varvec{\Phi }}^T \left( \mathbf {\widetilde{K}}^i {\varvec{\Phi }}{\varvec{\Delta \alpha }}^{i+1} + {\tilde{\mathbf {r}}}^i \right) = 0, \end{aligned}$$

where $\mathbf {\widetilde{K}}^i = \frac{\partial {\mathbf {f}}_{{\text {int}}}}{\partial \mathbf {u}}_{|{\bar{\mathbf {u}}} + {\varvec{\Phi }}(x) {\varvec{\alpha }}^i}$ is the tangent operator, ${\tilde{\mathbf {r}}}^i = {\mathbf {f}}_{{\text {int}}}({\bar{\mathbf {u}}} + {\varvec{\Phi }} {\varvec{\alpha }}^i)$ is the residual vector. It is important to recall that although the number of degrees of freedom of this system, *N*,  may be small, the cost of assembling the tangent operators and residuals remains expensive. The reduced model cannot be used “online” in this form, which is why an additional “system approximation” is necessary, which will be detailed in Sect. 3.1.2. For now, we will focus on our first proposition to construct a reduced space using the snapshot-POD methodology.

#### Snapshot POD

Once the snapshot is computed, an optimization problem can be solved to identify the reduced space that minimises a measure of the projection error of the samples. We define the snapshot matrix $\mathbf {S} = [ \mathbf {s}_1(t_1), \, \mathbf {s}_1(t_2), \ldots \mathbf {s}_1(t_{n_t}), \,\mathbf {s}_2(t_1), \ldots , \mathbf {s}_{n_{\mu }}(t_{n_t}) ],$ whose columns correspond to the computed samples in various far-field load cases over $n_t$ time steps.^6^

**Fig. 6:**
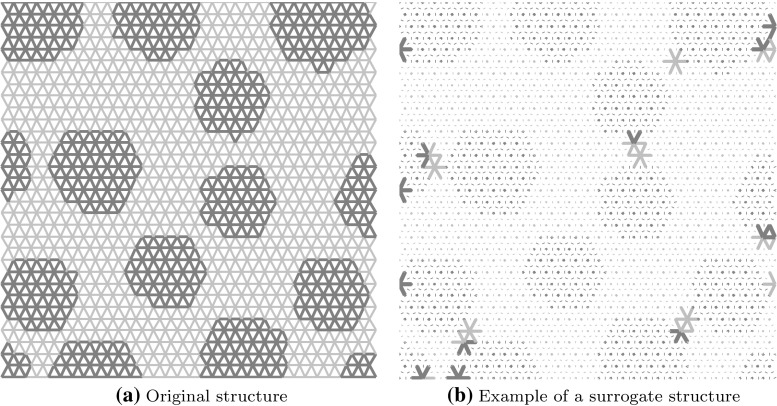
Example of a surrogate structure. The stiffness of the structure is evaluated on controlled elements only, while the other ones are just like ghosts

The POD minimisation problem reads:

14 $$\begin{aligned} {\left\{ \begin{array}{ll} \underset{{\varvec{\phi _1}},\ldots , {\varvec{\phi _l}}}{{\text {min}}} J_{\langle \cdot \rangle }^{\text {s}}\left( {\varvec{\phi _1}}, \ldots ,{\varvec{\phi _l}}\right) ,\\ \langle {\varvec{\phi _i}},\,{\varvec{\phi _j}} \rangle = \delta _{ij}, \end{array}\right. } \end{aligned}$$

where the scalar product $\langle \cdot \rangle$ remains to be defined and $\forall \mathbf {x},\,\Vert \mathbf {x} \Vert = \sqrt{\langle \mathbf {x},\,\mathbf {x} \rangle }.$ The cost function is defined as:

15 $$\begin{aligned} J_{\langle \cdot \rangle }^{\text {s}}\left( {\varvec{\phi _1}}, \ldots ,{\varvec{\phi _l}}\right) = \sum _{t_j=t_1}^{t_{n_t}} \sum _{i=1}^{n_\mu } \left\| \mathbf {s}_i\left( t_j\right) - \sum _{k=1}^{N} \left\langle \mathbf {\varvec{\phi }_k},\, \mathbf {s}_i\left( t_j\right) \right\rangle \mathbf {\varvec{\phi }_k}\right\| ^2.\nonumber \\ \end{aligned}$$

Now, we need to define the scalar product $\langle \cdot \rangle .$ The most common choice is the canonical scalar product (i.e., $\langle \mathbf {x},\,\mathbf {y}\rangle = \mathbf {x}^T\mathbf {y}$) which induces the ${\mathcal {L}}_2$-norm. In our case, the ${\mathcal {L}}_2$-norm of the displacement field has little interest. Since we are interested in the energy output of the RVE, we choose a scalar product induced by the initial structure stiffness $\mathbf {K_0}{\text {:}}\,\langle \mathbf {x},\,\mathbf {y}\rangle _{\mathbf {K_0}} = \mathbf {x}^T\mathbf {K_0}\mathbf {y}.$ This gives a structure specific measure of the displacement quantities. One can then show that solving (14) is equivalent to solve the eigenvalue problem:

16 $$\begin{aligned} \mathbf {S}\mathbf {S}^T\mathbf {K_0}{\varvec{\phi _i}} = \lambda _{i}{\varvec{\phi _i}}. \end{aligned}$$

This will provide a set of $\mathbf {K_0}$-orthogonal vectors that best represent the snapshot space in terms of elastic energy. We then have the following error (which represents how well the POD basis of order *N* approximates the snapshot $\mathbf {S}){\text {:}}$

17 $$\begin{aligned} \nu _{{\text {POD}}}\left( {\varvec{\phi _1}},\ldots , {\varvec{\phi _N}}\right) = \sqrt{\frac{\sum _{k = N+1}^{n_u} \lambda _{k}}{\sum _{k = 1}^{n_u} \lambda _{k}}}. \end{aligned}$$

#### System approximation

Constricting the displacement in a low-dimensional space does not provide a significant computational gain, even if the systems to be solved are of smaller dimension. This is because the material of study is nonlinear and history-dependent, and its stiffness varies not only in different areas of the material but also with time. This requires to evaluate the stiffness everywhere in the material and this at each time step of the simulation. This means that the numerical complexity remains despite the simplification on the displacement. Hence, to decrease the numerical complexity, thedomain itself needs to be approximated. Several authors have looked into that. Notable contributions include the hkyperreduction method [45], the missing point estimation [44], system approximation [32], DEIM [51] or more recently the energy-conserving and weighting method [47]. Those methods share the idea that the material properties will be evaluated only at a small set of points or elements within the material domain (Fig. 6). They differ in the way of selecting those points and in the treatment of that reduced information. In this paper, we will use the “gappy” method [52], very much like in [32, 51].

*Gappy method* The internal forces generated by the reduced displacement ${\mathbf {f}}_{{\text {int}}}({\varvec{\Phi }} {\varvec{\alpha }})$ 7 will be evaluated only in a small subset of the degrees of freedom ${\mathcal {I}}$ of the domain $\Omega .$ A procedure to select ${\mathcal {I}}$ will be described later on. All the elements in contact with those degrees of freedom have to be considered. We refer to those as the controlled elements. The internal forces will then be reconstructed by writing the internal forces as a linear combination of a few basis vectors themselves (just like it was made for the displacement).

18 $$\begin{aligned} {\mathbf {f}}_{{\text {int}}}({\varvec{\Phi }} {\varvec{\alpha }}) \approx \sum _1^{n_{{\text {gap}}}} \mathbf {\varvec{\psi }}_i \beta _i = \mathbf {\varvec{\Psi }} \mathbf {\varvec{\beta }}, \end{aligned}$$

where $[\mathbf {\varvec{\psi }_1}, \ldots , {\varvec{\psi }}_{{\mathbf {n}}_{{\text {gap}}}}] = {\varvec{\Psi }}$ is the forces basis of size $n_{{\text {gap}}}$ and ${\varvec{\beta }}$ the associated scalar coefficients.

The coefficients $\mathbf {\varvec{\beta }}$ of the expansion are found so that to minimise the norm of the difference between the linear expansion and the nonlinear term over the subset ${\mathcal {I}}{\text {:}}$

19 $$\begin{aligned} \underset{{\varvec{\beta }}^\star }{{\text {argmin}}} \left\| {\mathbf {f}}_{{\text {int}}}({\varvec{\Phi }} {\varvec{\alpha }}) -\mathbf {\varvec{\Psi }} \mathbf {\varvec{\beta }}^\star \right\| _\mathbf {P}, \end{aligned}$$

with $\mathbf {P}$ being a matrix so that $$\mathbf {P}ij = {\left\{ \begin{array}{ll} 1 \quad {\text {if}}\,i \in {\mathcal {I}} \quad {\text {and}} \,i = j, \\ 0 \quad {\text {otherwise}}, \end{array}\right. }$$ and $\Vert \mathbf {x} \Vert _\mathbf {P} = \Vert \mathbf {P}^T\mathbf {x} \mathbf {P}\Vert _2.\,\mathbf {P}$ can be written $\mathbf {E}\mathbf {E}^T$ with $\mathbf {E}$ being an extractor matrix so that $\mathbf {E}^T \mathbf {x}$ is the restriction of $\mathbf {x}$ to the set ${\mathcal {I}}.$ If the number of points in ${\mathcal {I}}$ is identical to the number of basis vectors $( {\varvec{\psi _i}})_{i=1,n_{{\text {gap}}}},\,\mathbf {\varvec{\beta }}^\star$ can be found by solving the equation:

20 $$\begin{aligned} \mathbf {E}^T \mathbf {\varvec{\Psi }} \mathbf {\varvec{\beta }} = \mathbf {E}^T{\mathbf {f}}_{{\text {int}}}({\varvec{\Phi }} {\varvec{\alpha }}), \end{aligned}$$

which implies:

21 $$\begin{aligned} \mathbf {\varvec{\beta }} = \left( \mathbf {E}^T \mathbf {\varvec{\Psi }}\right) ^{-1} \mathbf {E}^T {\mathbf {f}}_{{\text {int}}}({\varvec{\Phi }} {\varvec{\alpha }}), \end{aligned}$$

assuming $\mathbf {E}^T \mathbf {\varvec{\Psi }}$ is invertible. This assumption is true when using DEIM, since it insures the linear independence of the restriction of the basis to the reduced integration domain. In other strategies, such as hyperreduction, the size of the reduced integration domain may be chosen larger to guarantee well-posedness of the reduced equations.

At a Newton iteration of our POD-Galerkin framework, this reduces Eq. (13) to:

22 $$\begin{aligned} {\varvec{\Phi }}^T \mathbf {\varvec{\Psi }}\left( \mathbf {E}^T \mathbf {\varvec{\Psi }}\right) ^{-1} \mathbf {E}^T \mathbf {\widetilde{K}}^i {\varvec{\Phi }}\mathbf {\Delta \varvec{\alpha }} + {\varvec{\Phi }}^T \mathbf {\varvec{\Psi }}\left( \mathbf {E}^T \mathbf {\varvec{\Psi }}\right) ^{-1} \mathbf {E}^T \mathbf {\widetilde{r}}^i = \mathbf {0}.\nonumber \\ \end{aligned}$$

This can be rewritten in the form:

23 $$\begin{aligned} {\varvec{\Phi }}^T \mathbf {G} \mathbf {E}^T \mathbf {\widetilde{K}}^i {\varvec{\Phi }} \mathbf {\Delta \varvec{\alpha }} + {\varvec{\Phi }}^T \mathbf {G} \mathbf {E}^T \mathbf {\widetilde{r}}^i = \mathbf {0}, \end{aligned}$$

where we define the gappy operator $\mathbf {G} = \mathbf {\varvec{\Psi }}(\mathbf {E} \mathbf {\varvec{\Psi }})^{-1}.$

##### *Remark*

Note that once the “offline” stage operations are done, the bases $\mathbf {\varvec{\Phi }}$ and $\mathbf {\varvec{\Psi }}$ are calculated and the set of control points ${\mathcal {I}}$ is selected and the gappy operator is evaluated. In the “online” stage, all that remains to do is build a system of dimension equal to the size of the displacement basis and solve it which is computationally much cheaper. In particular, the evaluation of $\mathbf {K}$ will be substituted by the evaluation of $\mathbf {E}^T \mathbf {K},$ which allows great time savings.

*Selection of the controlled elements*: the selection of the control elements will be done using the DEIM [51]. This method finds a set of degrees of freedom ${\mathcal {I}}$ in a greedy manner from the internal forces basis $\mathbf {\varvec{\Psi }}.$ We briefly describe the method.

At iteration *j* of the greedy algorithm, $j-1$ points have been already selected. We define the extractor $\mathbf {E^j}$ that extracts those *j* selected degrees of freedom (i.e., for any vector $\mathbf {v},\,\mathbf {E^j}\mathbf {v}$ is a smaller vector containing only the *j* entries of $\mathbf {v}$ corresponding to the selected degrees of freedom). The residual ${\mathbf {r}}_{{\text {gap}}} = |\mathbf {\varvec{\psi }_{[1,j]}}\mathbf {\varvec{\beta }^j} - \mathbf {\varvec{\psi }_{j+1}}|$ is evaluated, where $\mathbf {\varvec{\psi }_{[1,j]}}$ is the matrix containing the first *j* vectors of the basis $\mathbf {\varvec{\Psi }}$ and $\mathbf {\varvec{\psi }_{j+1}}$ is the ${j+1}$th vectors in that basis. $\mathbf {\varvec{\beta }}$ is the solution of the minimisation problem

24 $$\begin{aligned} \mathbf {\varvec{\beta }} = \underset{{\varvec{\beta }}^\star }{{\text {argmin}}} \left\| \mathbf {E^j}\mathbf {\varvec{\psi }_{[1,j]}}\mathbf {\varvec{\beta }}^\star - \mathbf {E^j}\mathbf {\varvec{\psi }_{j+1}} \right\| _2. \end{aligned}$$

The solution is easily found: $\mathbf {\varvec{\beta }} = (\mathbf {E^j}\mathbf {\varvec{\psi }_{[1,j]}})^{-1}\mathbf {E^j}\mathbf {\varvec{\psi }_{j+1}}.$ The greedy procedure then selects the index of the highest entry in ${\mathbf {r}}_{{\text {gap}}}$ as the ${j+1}$th control degree of freedom. This procedure essentially selects the set of degrees of freedom that maximises the conditioning of the system (20). At the end of the greedy algorithm, the number of control degrees of freedom chosen equals the number of basis vectors $(\mathbf {\varvec{\psi }_i})_{n_{{\text {gap}}}}$ which makes system (20) well defined.

### A first “brute force” model reduction approach using snapshot POD on a snapshot randomly generated ensuring dissipation

In this section, we present the construction of a reduced model based on a random selection of the snapshot, constricting the random load paths to dissipate some energy of the structure each timestep. This is done to ensure the variability of the load paths so that maximum knowledge can be gained from the snapshot. In following, we show the method used to approximate the generation of such snapshots.

#### Random sampling of the parameter domain

To insure load paths that do not “turn back on themselves”, we enforce them to dissipate some energy in the structure at each timestep. The idea is that if no energy is dissipated, the structure will deform in an elastic manner, which will not add to the complexity of the snapshot space and will not be informative. We want the snapshot to be as varied as possible so that the reduced basis built from it can be exhaustive (in the sense that it is able to represent any solution resulting from any load path with a controlled error). Note that one could not put any dissipation constraint on the random load paths, but one would have to generate a much larger snapshot for it to statistically extend to the edges of the parameter space. Forcing dissipation saves computational time by computing only the most “informative” solutions.

To generate snapshots following this dissipation property, we will divide the load paths in increments, and enforce that at each increment, the maximum value of load path history is increased in either tension in $x,\,y$ or shear. This is an approximation, since this is not strictly equivalent to dissipating energy. However, this constraint is explicit, easy to implement, and provides essentially the extended snapshots we are looking for.

**Fig. 7:**
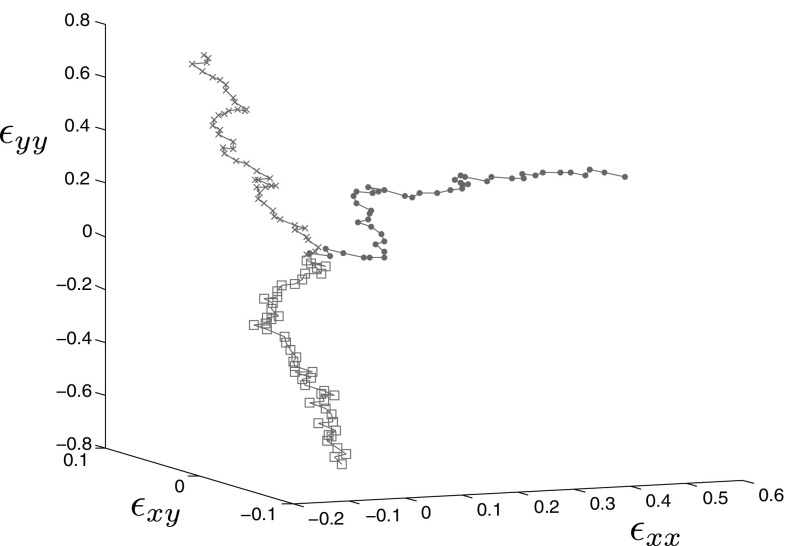
Example of loading paths obtained using the random procedure

In mathematical terms, the parameter space is sampled randomly by iteratively generating random load increments $${\varvec{\widetilde{\Delta \epsilon ^M}}}(t_n) = \begin{bmatrix} \widetilde{\Delta \epsilon _{xx}}(t_n)&\widetilde{\Delta \epsilon _{xy}}(t_n)\\ \widetilde{\Delta \epsilon _{xy}}(t_n)&\widetilde{\Delta \epsilon _{yy}}(t_n) \end{bmatrix}$$ of predefined norm $\Delta l.$ Initialising the loading path to be generated by ${\varvec{\epsilon ^M}}(t_0) = {\varvec{0}},$ the path is iteratively incremented as:

25 $$\begin{aligned} {\varvec{\epsilon ^M}}\left( t_{n+1}\right) = {\varvec{\epsilon ^M}}\left( t_n\right) + {\varvec{\widetilde{\Delta \epsilon ^M}}}\left( t_n\right) , \quad {\text {with}}\, \left\| {\varvec{\widetilde{\Delta \epsilon ^M}}}\left( t_n\right) \right\| = \Delta l,\nonumber \\ \end{aligned}$$

where $\Vert {\varvec{\widetilde{\Delta \epsilon ^M}}}(t_n)\Vert = \sqrt{\widetilde{\Delta \epsilon _{xx}}^2(t_n) + \widetilde{\Delta \epsilon _{yy}}^2(t_n) + \widetilde{\Delta \epsilon _{xy}}^2(t_n)}.$ The random load increments ${\varvec{\widetilde{\Delta \epsilon ^M}}}(t_n)$ are forced to create dissipation, by ensuring that at least one of the following inequalities is true at each timestep:

26 $$\begin{aligned}&\left\langle \widetilde{\Delta \epsilon _{xx}}\left( t_n\right) \right\rangle ^{+} > \max _{k \in \llbracket 0, n-1 \rrbracket } \widetilde{\Delta \epsilon _{xx}}\left( t_k\right) ,\end{aligned}$$

27 $$\begin{aligned}&\left\langle \widetilde{\Delta \epsilon _{yy}}\left( t_n\right) \right\rangle ^{+} > \max _{k \in \llbracket 0, n-1 \rrbracket } \widetilde{\Delta \epsilon _{yy}}\left( t_k\right) ,\end{aligned}$$

28 $$\begin{aligned}&\left| \widetilde{\Delta \epsilon _{xy}}\left( t_n\right) \right| > \max _{k \in \llbracket 0, n-1 \rrbracket } \left| \widetilde{\Delta \epsilon _{xy}}\left( t_k\right) \right| , \end{aligned}$$

where $\langle x \rangle ^{+}$ is the positive part of *x*. These conditions mean that either the tension in *x* direction, in *y* direction or shear has to increase at each timestep. When no dissipation is created, the damage law behave essentially linearly and do not add to the complexity of the snapshot space. An example of a few loading paths generated using this method is displayed in Fig. 7. The randomness of this procedure will allow to explore the parameter space exhaustively, as long as the number of paths generated is large enough.

To improve the reliability of the procedure, the quality of the ROM could be tested by evaluating the error over some random validation set $\Xi _{{\text {test}}}$ different from the snapshot set $\Xi .$ If the average error over that set is larger than some tolerance, the initial snapshot could be enriched iteratively until that tolerance is achieved. This is described in pseudo-code in Algorithm 2. This strategy will not be developed further in this paper.
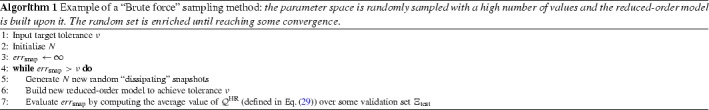


#### Application of the random snapshot-POD procedure and numerical findings

**Fig. 8:**
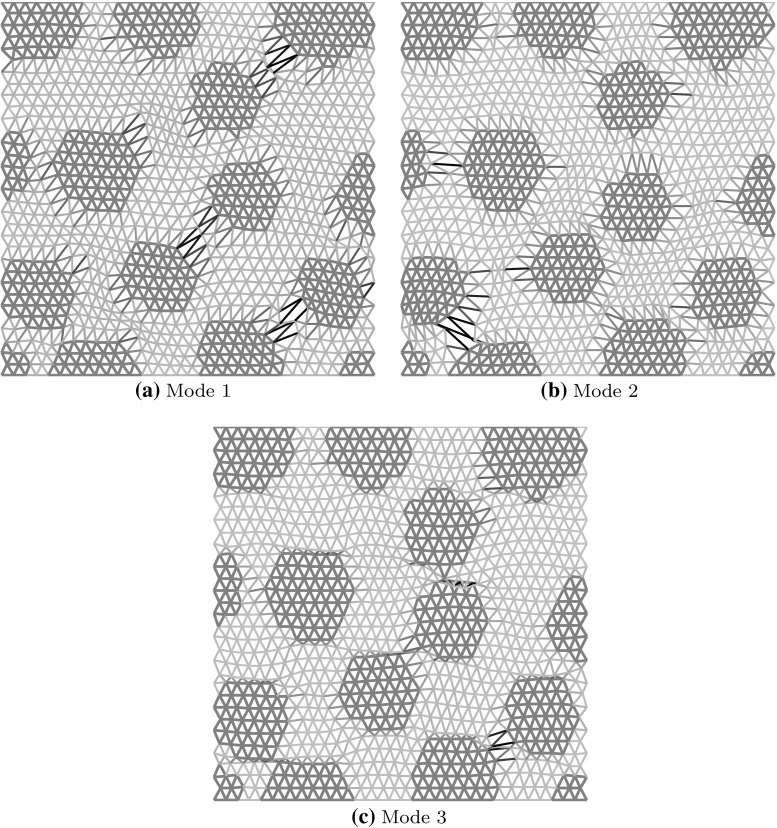
Vectors associated to the three largest eigenvalues obtained using the snapshot-POD procedure with random snapshot selection. *Darker bars* indicates larger damage. The damage localises between pairs of inclusions

*Displacement basis*: we proceed to apply the snapshot-POD procedure with random snapshot selection described in Sect. 3.2. 36 Load paths are randomly generated. The first few vectors of the POD expansion are displayed in Fig. 8.

*System approximation*: we follow the procedure described in 3.1.2. The basis $\mathbf {\varvec{\Psi }}$ is extracted from the snapshot space generated by the same loading paths used for the displacement basis $\mathbf {\varvec{\Phi }}.$ The set of controlled elements is selected using the DEIM [51]. The amount of vectors in the basis $\mathbf {\varvec{\Psi }}$ is chosen so that the error generated by the system approximation is of the same order than the global error of the ROM.

To this purpose we define the quantity of interest ${\mathcal {Q}}$ as the norm of the error. More specifically, we denote ${\mathcal {Q}}^{{\text {R}}}$ the average norm over time of the error between exact and reduced-order solution using no hyperreduction and ${\mathcal {Q}}^{{\text {HR}}}$ the average norm over time of the error between exact and “hyperreduced-order solution” actually using the hyperreduction:

29 $$\begin{aligned}&{\mathcal {Q}^{{\text {R}}}(\mu )}^2 = \sum _{t = t_0}^{t_{n_t}}\frac{\Vert \mathbf {u}(\mu ,\,t) - \mathbf {u}^{{\text {R}}}(\mu ,\,t)\Vert ^2_{\mathbf {K_0}}}{n_t+1}\quad {\text {and}}\quad \nonumber \\&{{\mathcal {Q}}^{{\text {HR}}}(\mu )}^2= \sum _{t = t_0}^{t_{n_t}}\frac{\Vert \mathbf {u}(\mu ,\,t) - \mathbf {u}^{{\text {HR}}}(\mu ,\,t)\Vert ^2_{\mathbf {K_0}}}{n_t+1}, \end{aligned}$$

with $\mathbf {u}(t)$ the exact solution, $\mathbf {u}^{{\text {R}}}(\mu ,\,t;\,\mathbf {\varvec{\Phi }}),$ the reduced-order solution without the system approximation using the displacement basis $\mathbf {\varvec{\Phi }},$ and ${\mathbf {u}}^{{\text {HR}}}(\mu ,\,t;\,\mathbf {\varvec{\Phi }},\, \mathbf {\varvec{\Psi }})$ the complete ROM with system approximation using the displacement basis $\mathbf {\varvec{\Phi }}$ and the static basis $\mathbf {\varvec{\Psi }}.$ Note that we skip the dependency of the solution on the bases $\mathbf {\varvec{\Phi }}$ and $\mathbf {\varvec{\Psi }}$ in the following for simplicity of the notations. ${{\mathcal {Q}}^{{\text {HR}}}(\mu )}^2$ can then be decomposed in the following way:

30 $$\begin{aligned} {{\mathcal {Q}}^{{\text {HR}}}(\mu )}^2&= \sum _{t = t_0}^{t_{n_t}}\frac{\Vert \mathbf {u}(\mu ,\,t) - \mathbf {u}^{{\text {R}}}(\mu ,\,t) +\mathbf {u}^{{\text {R}}}(\mu ,\,t) - \mathbf {u}^{{\text {HR}}}(\mu ,\,t)\Vert ^2_{\mathbf {K_0}}}{n_t+1} \end{aligned}$$

31 $$\begin{aligned}&\le {{\mathcal {Q}}^{{\text {R}}}(\mu )}^2 + \underbrace{\sum _{t = t_0}^{t_{n_t}}\frac{\Vert \mathbf {u}^{{\text {R}}}(\mu ,\,t) - \mathbf {u}^{{\text {HR}}}(\mu ,\,t)\Vert ^2_{\mathbf {K_0}}}{n_t+1}}_{ \mathop {=}\limits ^{def} \widetilde{{\mathcal {Q}}^{{\text {HR}}}(\mu )}^2}. \end{aligned}$$

Taking this in consideration, the basis $\mathbf {\varvec{\Psi }}$ is chosen to be the smallest (i.e., the one with the least amount of vectors) that verifies the inequality:

32 $$\begin{aligned} \widetilde{{\mathcal {Q}}^{{\text {HR}}}(\mu )} \le {\mathcal {Q}}^{{\text {R}}}(\mu ). \end{aligned}$$

**Fig. 9:**
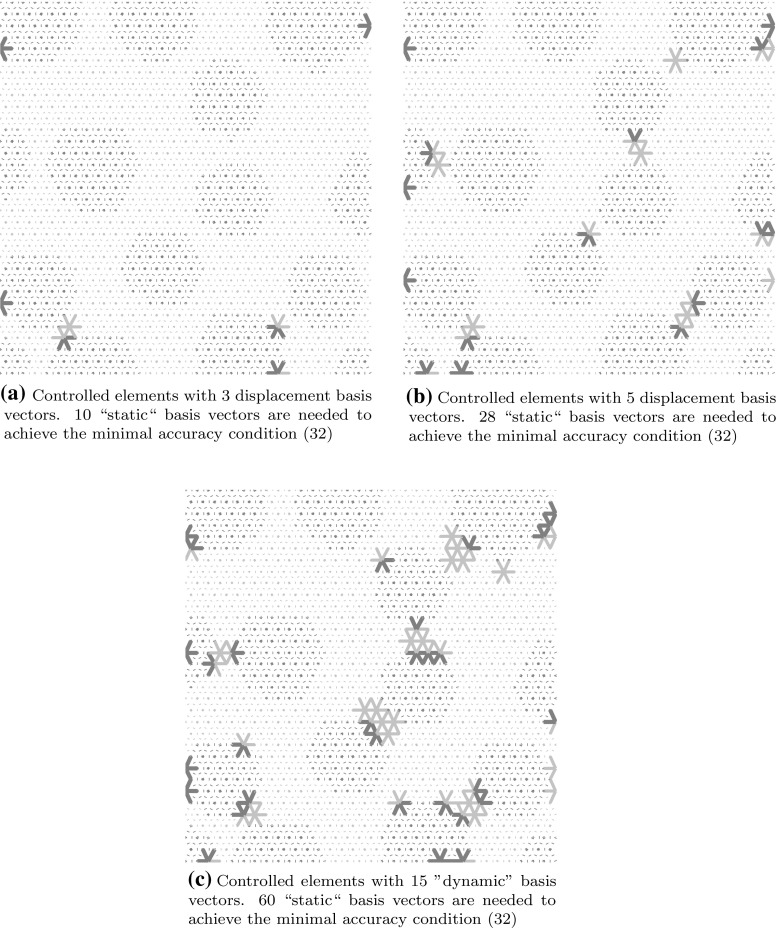
Controlled elements selected using various basis sizes. The larger the basis, the more controlled elements are needed. The elements tend to gather around the regions where the variation of displacement is the highest, hence where the variation of the internal forces will be high

**Fig. 10:**
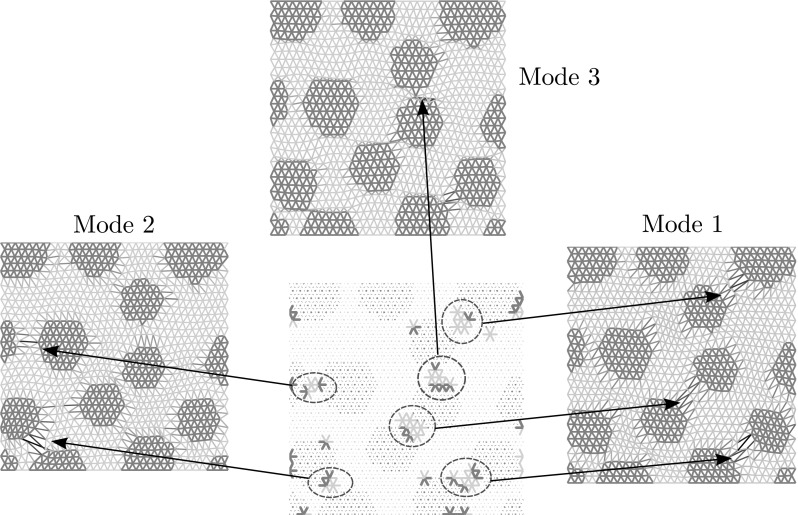
Regions of interest selected by the system approximation procedure. Those regions (it circled in the figure) are matching the areas of higher displacement found in the POD bases. This is intuitively good, since those elements have to give enough information to be able to reconstruct the internal forces over the entire domain. Those are the elements whose behaviour vary the most when changing the loading path (which is the parameter of the reduced model), hence containing the core information necessary to build up an accurate reconstruction

This guarantees that the error generated by the system approximation is controlled by the error generated by approximating the displacement. The location of controlled elements (which are all the elements in contact with the control degrees of freedom) is shown in Fig. 9 for various basis sizes. It is interesting to remark that the controlled elements gather around inclusions where damage is the highest. Figure 10 illustrate this effect.

##### *Remark*

Note that in Eq. (31), we defined the quantity $\widetilde{{\mathcal {Q}}^{{\text {HR}}}(\mu )}$ which defines the error between the reduced and the hyperreduced model which is different from ${\mathcal {Q}}^{{\text {HR}}}(\mu ),$ which defines the error between the exact solution and the hyperreduced model.

*Numerical savings* in this section, we will test the performance of the method by comparing the relative error between the “truth” solution of the RVE problem, which is the solution obtained when using the full order model, and the ROM.

The following load path considered for testing the efficiency of the model is set using the following effective strain: $${\varvec{\epsilon }}^{\mathbf{M}}(t) = \frac{t}{T} \cdot \begin{bmatrix} 1&1\\ 1&1 \end{bmatrix}.$$ Note that this case is not in the snapshot set.

**Fig. 11:**
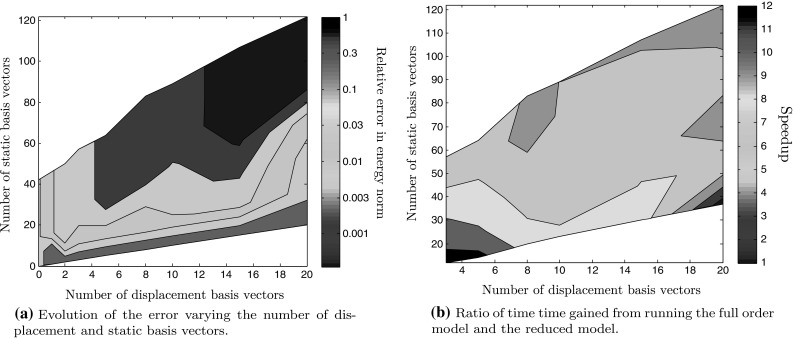
Numerical results tested on a loading path not included in the snapshot space. Here, the snapshot selection was arbitrary and relatively fine which allows to consider various number of basis sizes. Note that one could use these plots as a way to determine the sizes of the bases to maximise the speedup for aspecific target error

We then proceed to solve the RVE boundary value problem subjected to this loading path using both the full order model and the ROM while varying the sizes of the displacement and static bases. Induced errors and times gained are displayed in Fig. 11.

Several remarks can be made:

- As expected, the error decreases when the number of either the displacement or static bases vectors increases. A higher dimensional representation of the solution leads unsurprisingly to more accuracy.
- The time gained using the reduced model becomes more and more important when the number of vectors in the bases decreases.
- Looking at Fig. 11b, it can be seen that the speedup is roughly dependent on the size of the static bases, rather than on the displacement basis. Indeed, the number of controlled elements, which is linked to the amount of computations to be done, is directly linked to the dimension of the static basis ${\varvec{\Psi }}.$
- To have a well defined ROM, the dimension of the static basis ${\varvec{\Psi }}$ should at least match the dimension of the displacement basis ${\varvec{\Phi }}.$ However, it can be seen that to achieve a reasonable tolerance on the error, the dimension of the static basis should actually be relatively larger.

**Fig. 12:**
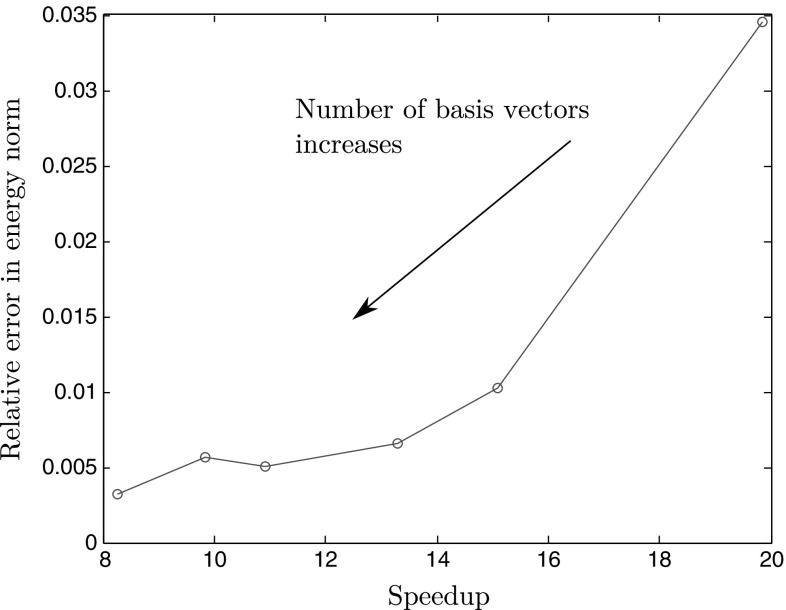
Evolution of the error with respect to the speedup while increasing the number of basis vectors. For each size of the displacement basis ${\varvec{\Phi }},$ the number of vectors in the static basis ${\varvec{\Psi }}$ is chosen according to the rule defined in Eq. (32)

The error with respect to the speedup for a range of reduced space sizes is displayed in Fig. 12. What we call speedup here is the ratio of the elapsed time of the full order simulation over the elapsed time of the reduced model. It represents how many times faster is the ROM compared to the full order model.

It can be seen that there is a proportional relation between speedup and error: as the number of basis functions increases, the speedup and the error decrease. The user can reduce the error at the price of having a slower simulation. What makes the reduced model faster is purely the bypassing of most of the elements when computing the internal forces or the tangent stiffness (this bypassing is possible thanks to the system approximation technique). Note that the speedup is not purely equal to the ratio between controlled elements and total number of elements since the NR procedure requires more steps to converge in the ROM scheme than in the full order model. Another remark is that beyond a certain dimension of the reduced space, the error does not decrease very much and reaches a plateau. This means that no matter how many vectors in the basis, a maximum accuracy is achieved. This can be explained by the fact that the loading path tested is not part of the snapshot. The only way to decrease this residual error is to enrich the snapshot space. Let us define ${\mathbf {u}}_{{\text {snap}}}(t)$ as the projection of the exact solution onto the snapshot space. Using the same principle than Eq. (31), we can decompose the error further (dropping parameters for clarity):

33 $$\begin{aligned} {{\mathcal {Q}}^{{\text {HR}}}}^2&= \sum _{t = t_0}^{t_{n_t}}\frac{\Vert \mathbf {u}(t) - {\mathbf {u}}_{{\text {snap}}}(t) +{\mathbf {u}}_{{\text {snap}}}(t) -\mathbf {u}^{\text {R}}(t) + \mathbf {u}^{{\text {R}}}(t) - \mathbf {u}^{{\text {HR}}}(t) \Vert ^2_{\mathbf {K_0}}}{n_t+1} \end{aligned}$$

34 $$\begin{aligned}&\le \sum _{t = t_0}^{t_{n_t}}\frac{\Vert \mathbf {u}(t) - {\mathbf {u}}_{{\text {snap}}}(t) \Vert ^2_{\mathbf {K_0}}}{n_t+1} + \sum _{t =t_0}^{t_{n_t}}\frac{\Vert {\mathbf {u}}_{{\text {snap}}}(t) -\mathbf {u}^{{\text {R}}}(t) \Vert ^2_{\mathbf {K_0}}}{n_t+1} +\widetilde{{\mathcal {Q}}^{{\text {HR}}}}^2. \end{aligned}$$

$\sum _{t = t_0}^{t_{n_t}}\frac{\Vert {\mathbf {u}}_{{\text {snap}}}(t) - \mathbf {u}^{{\text {R}}}(t)\Vert ^2_{\mathbf {K_0}}}{n_t+1}$ and $\widetilde{{\mathcal {Q}}^{{\text {HR}}}}^2$ can be made as small as desired by taking high dimensional bases $\mathbf {\varvec{\Phi }}$ and $\mathbf {\varvec{\Psi }}.$ The residual error that remains is $\sum _{t = t_0}^{t_{n_t}}\frac{\Vert \mathbf {u}(t) - {\mathbf {u}}_{{\text {snap}}}(t)\Vert ^2_{\mathbf {K_0}}}{n_t+1},$ which entirely depends upon the richness of the snapshot space.

**Fig. 13:**
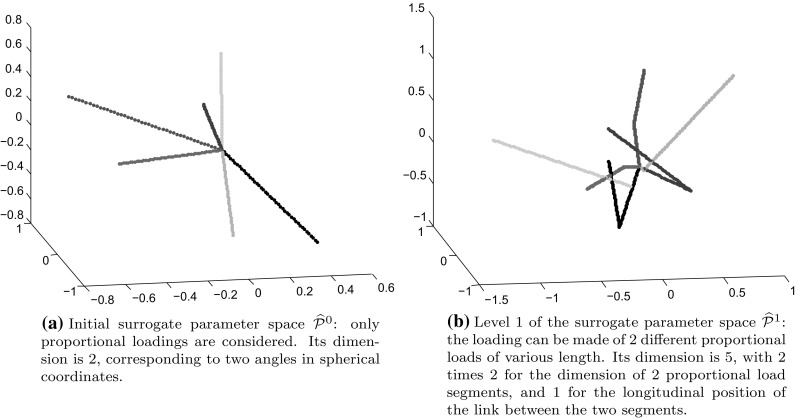
Examples of loadings paths for levels 0 and 1 of the surrogate parameter spaces

We will deal with this issue in the next section by using a Bayesian-optimized snapshot selection which will allow to guess the error between the discrete solutions computed for the snapshot set.

### Model reduction using a POD-greedy algorithm based on a Bayesian-optimized snapshot selection designed for dealing with high-dimensional parameter spaces

As said in the previous section, it may not be satisfactory to use an arbitrary sampling method, since some important information could be unwittingly dropped out. The accuracy of the reduced model greatly depends on the snapshot space and how well it samples the parameter space. Here, the parameter space contains any load path (based on the macro-strain ${\varvec{\epsilon ^M}}(t)$) over a certain period of time until ellipticity of the mechanical problem is lost. After time discretisation, the parameter space is of dimension $2 \times n_{{\text {t}}},$ since in two dimensions the load can be uniaxial in the *x* or *y* direction or in shear, and we set a fixed load increment norm between two timesteps. $n_{{\text {t}}}$ stands for the number of time steps required to reach fracture.

This section attempts to address the problem of ensuring that the snapshot space is sufficiently fine so that a reduced model of sufficient accuracy can be built upon it. Given the high dimension of the parameter space, its effective sampling is based on a combination of three necessary cost-effective strategies:

- First, the high-dimensional parameter space $\mathcal {P}$ is restricted to a hierarchical sequence of much lower dimensional pseudo-parameter spaces $\widehat{{\mathcal {P}}}^n$ which enable to avoid the “curse of dimensionality”. Starting from a pseudo-parameter space $\widehat{{\mathcal {P}}}^0$ containing proportional loadings only, it is iteratively refined until reaching some “convergence”. This approach is described in Sect. 3.3.1.
- Second, within each pseudo-parameter spaces $\widehat{\mathcal {P}}^n,$ rather than a random and fine sampling typically used in traditional POD-greedy approaches, an effective selection procedure allowing few evaluations of an error indicator is done using a Gaussian process predictor. This strategy is explained in Sect. 3.3.2.
- A statistical correspondence between the error indicator and the true error is built using Gaussian process regression to control the convergence of the procedure. This is described in Sect. 3.3.3.

#### Definition of a sequence of surrogate parameter spaces of low dimension

We would like to sample the parameter space exhaustively. To this purpose, the use of Gaussian process methods [42, 53] are attractive as they provide an excellent predictor as well as statistical information that allows to trust the model. However, it is almost inapplicable in a high-dimensional context, as it requires a decent amount of data in proportion with the dimensionality of the phenomenon to study. To circumvent the curse of dimensionality, we propose to define a sequence of low-dimensional surrogate parameter spaces of progressively finer dimension $\widehat{{\mathcal {P}}}^i.$ The initial surrogate space $\widehat{{\mathcal {P}}}^0$ contains all proportional loadings only (i.e., the loading path is a straight line). This space only is of dimension 2 (well-defined by two angles when considering spherical coordinates). The surrogate space $\widehat{{\mathcal {P}}}^1$ is defined as two successive proportional loadings with different directions (Fig. 13). This space has five dimensions since two dimensions can be counted for each proportional part of the loading plus one more dimension for the length of the load at which the second proportional loading starts. This sequence can carry on with space *n*,  the space of loads with $n+1$ distinct proportional loadings, which has dimension $2\times n + (n-1).$ We also have the property that surrogate space *n* is included in surrogate space $n+1{\text {:}}$

35 $$\begin{aligned} \forall n \in {\mathbb {N}}, \quad \widehat{{\mathcal {P}}}^n \subset \widehat{{\mathcal {P}}}^{n+1}. \end{aligned}$$

Now, with such an inclusive decomposition of the parameter space, it becomes possible to infer what level of refinement is necessary to consider, for building an accurate reduced model. Indeed, a ROM can be constructed based on snapshots from surrogate parameter space $\widehat{\mathcal {P}}^n,$ and if it represents well any solution in parameter space $\widehat{\mathcal {P}}^{n+1}$ (which is a space with a finer discretisation of the loading paths ${\varvec{\epsilon }}^M$), we can assume that the current reduced model is satisfactory and there is no need to consider finer parameter spaces. The procedure is described in Algorithm 2.
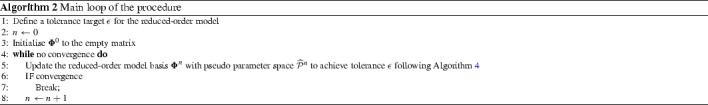


#### Exhaustive sampling of the surrogate parameter spaces using a Gaussian process predictor

In this section, given a dimension for the surrogate parameter space, we are looking for the value of the parameter leading to the highest error between the exact solution and the solution computed using our reduced model.

*Standard POD-greedy procedure* in traditional POD-greedy strategies [40], an a posteriori error bound $\Delta ^k(\mu )$ inexpensive to compute is assumed available, which allows for the estimation of the error between the full order model and the ROM at step *k* of the procedure, on a fine discretisation $\Xi \in {\mathcal {P}}$ of the parameter space. At step *k* of the procedure, the full order model is evaluated at the parameter value $\mu _{{\text {max}}}^k$ satisfying:

36 $$\begin{aligned} \mu _{{\text {max}}}^k = \underset{\mu \in \Xi }{{\text {argmax}}} \Delta ^k(\mu ). \end{aligned}$$

The ROM is updated with this new information and the algorithm carries on until reaching some tolerance.

In practice, error bounds $\Delta ^k(\mu )$ are available for linear problems. In the general nonlinear case, no sharp error bound is available, and one has to rely on an error indicator at the parameter value $\mu$ instead: ${\mathcal {J}}(\mu ).$ This error indicator, does not provide a bound on the error, but rather a measure of its magnitude. Though less expensive than computing the exact solution at $\mu ,$ we will see in the next section that the evaluation of this indicator at all values of a fine discretisation of the parameter space is not affordable. To alleviate this issue, the error indicator surface over the parameter space will be approximated using a Gaussian process predictor to allow for only a few, well chosen, evaluations of the indicator.

*Definition of an error indicator based on the residual* in our case the quantity of interest is the error ${\mathcal {Q}}^{{\text {HR}}}$ defined in Eq. (29). Having no rigorous error bound at hand, we will use an error indicator instead. The norm of the residual $\mathbf {r}(t_k,\,\mu ;\,{\varvec{\Phi }},\,{\varvec{\Psi }})$ can be used as such an indicator at each timestep (as described in [43, 54]). Here the residual at timestep $t_k$ is defined as:

37 $$\begin{aligned} \mathbf {r}\left( t_k,\,\mu ;\,{\varvec{\Phi }},\,{\varvec{\Psi }}\right) = {\mathbf {f}}_{{\text {int}}}\left( {\bar{\mathbf {u}}}\left( t_k;\, \mu \right) + {\varvec{\Phi }} {\varvec{\alpha }}\left( t_k;\,\mu \right) \right) , \end{aligned}$$

where ${\varvec{\alpha }}(t_k;\,\mu )$ is the converged solution at $t = t_k$ obtained from the Newton procedure described in Eq. (23).

Since we would like an error indicator that is taking into account the entire time-history of the solution, we define a time-independent norm of the residual for the complete ROM (i.e., including both approximation of the displacement in a low-dimensional space and approximation of the internal forces):

38 $$\begin{aligned} {\mathcal {R}}(\mu ;\, {\varvec{\Phi }},\, {\varvec{\Psi }}) = \sqrt{\frac{\sum _{k=0}^{k=N} \Vert \mathbf {r}(t_k,\, \mu ;\, {\varvec{\Phi }},\, {\varvec{\Psi }})\Vert ^2}{N+1}}. \end{aligned}$$

##### *Remark*

Note that the residual ${\mathcal {R}}$ will almost always not be null. Indeed, what is solved in the reduced model leads to the cancellation, at each timestep, of the projected residual:${\varvec{\Phi }}^T \mathbf {G} \mathbf {E}^T{\mathbf {f}}_{{\text {int}}}(\mathbf {\bar{u}}(t_k;\,V{\varvec{\epsilon }^M}) + {\varvec{\Phi }} {\varvec{\alpha }}(t_k;\,\mathbf {\varvec{\epsilon }^M})),$ using Newton iterations.

Now, since the residual is defined on the fine discretisation of the domain, the cost of its evaluation presents still a significant cost. This can be partly alleviated by evaluating a surrogate $\widetilde{{\mathcal {R}}}$ of the global residual ${\mathcal {R}}$ over only a subset of the timesteps. We then define the error indicator:

39 $$\begin{aligned}&{\mathcal {J}}(\mu ;\, {\varvec{\Phi }},\, {\varvec{\Psi }})\nonumber \\&\quad = \widetilde{{\mathcal {R}}}(\mu ;\, {\varvec{\Phi }},\, {\varvec{\Psi }})= \sqrt{\frac{\sum _{t_k \in \widetilde{{\mathcal {T}}}} \Vert \mathbf {r}(t_k,\, \mu ;\, {\varvec{\Phi }},\, {\varvec{\Psi }})\Vert ^2}{|\widetilde{{\mathcal {T}}}|}}, \end{aligned}$$

where $\widetilde{{\mathcal {T}}}$ is a subset of the time discretisation used to compute the simulation; for example $\widetilde{{\mathcal {T}}}$ may contain only one in every five timesteps. In Sect. 3.3.4, we may refer to ${\mathcal {J}}(\mu ;\, {\varvec{\Phi }},\, {\varvec{\Psi }})$ as ${\mathcal {J}}^{{\text {R}}}$ or ${\mathcal {J}}^{{\text {HR}}}$ depending on if we are considering the residual of the ROM without or with hyperreduction, respectively. The main advantage is that it can provide an indicator of the magnitude of the error for various values of the parameter $\mu$ (which in our problem is the far-field strain ${\varvec{\epsilon ^M}}$) at a much cheaper cost that having to evaluate both the exact and reduced solution. Nevertheless ${\mathcal {J}}$ remains a non-negligible quantity to compute and we show in the next paragraph how to exhaustively explore the parameter space despite a limitation on the number of evaluation of this error indicator and hence of that residual.

*Gaussian process regression of the residual surface for efficient evaluation of the error* in traditional POD-greedy procedures, a discrete set $\Xi \in {\mathcal {P}}^n$ is built arbitrarily to sample the parameter space. It is typically very fine. The goal of this section is to define a set $\Xi$ that is of relatively small cardinality but is chosen so that it is likely to contain the values of the parameter leading to the highest error. To this purpose, we follow a procedure similar to the one described in [42, 43].

The first ingredient is Gaussian process regression [53] (also called kriging in the literature): starting from an initial set $\Xi _0$ chosen randomly containing few values of the parameter and an associated set of values of the error indicator $\{{\mathcal {J}}(\mu _i) |\mu _i \in \Xi _0 \},$ a Gaussian process regression approximating the error indicator ${\mathcal {J}}$ with a confidence interval over the entire parameter domain ${\mathcal {P}}^n,$ will be constructed for each step *m* of the sampling process. This regression will be used to iteratively enrich $\Xi _m$ with values of the parameter ${\varvec{\mu }}_m$ where the probability of having large values of the error indicator ${\mathcal {J}}$ is the highest.

The method is based on the assumption that the data studied is following a joint Gaussian distribution defined by a mean ${\bar{\mathbf {m}}},$ which can be unknown, and a covariance matrix, whose shape is defined a priori by the user. A common covariance function is the squared exponential:

40 $$\begin{aligned} cov\left( \mathbf {x^p},\,\mathbf {x^q}\right) = \sigma ^2 \exp \left( \left( \mathbf {x^p} - \mathbf {x^q}\right) ^T \mathbf {I}_{\varvec{\theta }} \left( \mathbf {x^p} - \mathbf {x^q}\right) \right) , \end{aligned}$$

with $\mathbf {I}_{\varvec{\theta }}$ being a diagonal matrix with diagonal element $\theta _i$ on the *i*th row. For the observations at parameter values $\Xi$ (of cardinality *N*), ${\mathcal {J}}$ follows the joint Gaussian distribution of mean ${\bar{\mathbf {m}}}$ and covariance $\mathbf {Cov}(\Xi ,\,\Xi ){\text {:}}$

41 $$\begin{aligned}&{\mathcal {J}}_{\bar{\mathbf {m}},\sigma ^2,{\varvec{\theta }}}(\mathbf {x}) \sim \frac{1}{(2\pi )^{N/2}{|\mathbf {Cov}(\Xi ,\,\Xi )|}^{1/2}}\nonumber \\&\quad e^{-\frac{1}{2}(\mathbf {x}- \bar{\mathbf {m}})(\mathbf {Cov}(\Xi ,\,\Xi ))^{-1}(\mathbf {x} - \bar{\mathbf {m}})}, \end{aligned}$$

or more simply written:

42 $$\begin{aligned} {\mathcal {J}} \sim {\mathcal {N}}({\bar{\mathbf {m}}},\,\mathbf {Cov}(\Xi ,\,\Xi )), \end{aligned}$$

where the element in *i*th row and *j*th column of $\mathbf {Cov}(\Xi ,\,\Xi )$ is $\{\mathbf {Cov}_{ij}\} = cov(\mathbf {x^i},\,\mathbf {x^j})$ with $\mathbf {x^i},\,\mathbf {x^j} \in \Xi .$ $\bar{\mathbf {m}},\,{\varvec{\theta }}$ and $\sigma ^2$ are hyperparameters that need to be determined. Assuming the knowledge of the data $\Xi ,$ it is done through the maximisation of the likelihood function:

43 $$\begin{aligned}&{\mathcal {L}}_\Xi \left( {\bar{\mathbf {m}},\,\sigma ^2,\,{\varvec{\theta }}}\right) =\frac{1}{(2\pi )^{N/2}{|\mathbf {Cov}(\Xi ,\,\Xi )|}^{1/2}}\nonumber \\&\quad e^{-\frac{1}{2}(\mathbf {x}- \bar{\mathbf {m}})(\mathbf {Cov}(\Xi ,\,\Xi ))^{-1}(\mathbf {x} - \bar{\mathbf {m}})}. \end{aligned}$$

This maximization aims to make the Gaussian process distribution we are constructing as consistent as possible with the data at hand. Poorly chosen values of the hyperparameters will lead to poor predictions. This is typically the case with little data at hand but improves as more and more samples are computed. See [53] or [42] for more details on the determination of the hyperparameters.

Then, adding test inputs, we have (with the input parameters $\Xi$ carrying a subscript $\star$ being the test inputs, the other ones being the training inputs):

44 $$\begin{aligned} \begin{bmatrix} {\mathcal {J}} \\ {\mathcal {J}}_\star \end{bmatrix} \sim {\mathcal {N}}\left( \bar{m},\, \begin{bmatrix} \mathbf {Cov}(\Xi ,\,\Xi )&\mathbf {Cov}(\Xi ,\,\Xi _\star )\\ \mathbf {Cov}(\Xi _\star ,\,\Xi )&\mathbf {Cov}(\Xi _\star ,\,\Xi _\star ) \end{bmatrix} \right) . \end{aligned}$$

This distribution makes no use of the data we have at end. To obtain the posterior distribution which actually uses the knowledge of the data points, one can condition the prior distribution to the observations and obtain the distribution:

45 $$\begin{aligned}&{\mathcal {J}}_\star |\Xi _\star ,\,\Xi ,\,{\mathcal {J}} \sim {\mathcal {N}}\left( \bar{m} + \mathbf {Cov}(\Xi _\star ,\,\Xi )\mathbf {Cov}(\Xi ,\,\Xi )^{-1}({\mathcal {J}} -\bar{m}),\right. \end{aligned}$$

46 $$\begin{aligned}&\left. \mathbf {Cov}(\Xi _\star ,\,\Xi _\star ) - \mathbf {Cov}(\Xi _\star ,\,\Xi )\mathbf {Cov}(\Xi ,\,\Xi )^{-1}\mathbf {Cov}(\Xi ,\,\Xi _\star ) \right) . \end{aligned}$$

From this expression one can deduce a predictor at any parameter value $\mu$ by taking the mean value,

47 $$\begin{aligned} {\mathcal {J}}^m_\star (\mu ) = \bar{m} + \mathbf {Cov}(\{ \mu \},\,\Xi )\mathbf {Cov}(\Xi ,\,\Xi )^{-1}({\mathcal {J}} - \bar{m}), \end{aligned}$$

together with a standard error, using the covariance:

48 $$\begin{aligned} s(\mu )^2= & {} \mathbf {Cov}(\{ \mu \},\,\{ \mu \}) - \mathbf {Cov}(\{ \mu \},\,\Xi )\mathbf {Cov}(\Xi ,\,\Xi )^{-1}\nonumber \\&\mathbf {Cov}(\Xi ,\,\{ \mu \}). \end{aligned}$$

Using this regression at step *m* of the sampling process, the maximum value ${{\mathcal {J}}^m_\star }_{{\text {max}}}$ of the current Gaussian predictor ${\mathcal {J}}^m_\star$ over the parameter domain is computed. Rather than considering the parameter value achieving this maximum as a new test point, the procedure searches for the parameter value that has the highest probability of improving that maximum value by some predefined percentage, which defines a target value $T({{\mathcal {J}}^m_\star }_{{\text {max}}}).$ Indeed, this allows to take into account the trade-off between maximal value of the predictor and uncertainty characterized by the standard error. Since ${\mathcal {J}}_\star (\mu )$ has a normal distribution with mean ${\mathcal {J}}^m_\star (\mu )$ and standard error $s(\mu ),$ the probability of improvement of ${\mathcal {J}}_\star (\mu )$ beyond the target $T({{\mathcal {J}}^m_\star }_{{\text {max}}})$ is:

49 $$\begin{aligned} \pi \left( {\varvec{\epsilon ^M}};\, T\right) = \varphi \left( \frac{{\mathcal {J}}^m_\star (\mu ) - T({{\mathcal {J}}_\star }_{{\text {max}}})}{s(\mu )} \right) , \end{aligned}$$

where $\varphi$ is the normal cumulative distribution function. The parameter $\mu _m$ maximising this probability is then added to the set $\Xi _{m-1},$ creating the new set $\Xi _{m}$ that will be used to build the Gaussian predictor for the next step (with new values of the hyperparameters $\bar{\mathbf {m}},\, \sigma ,\,{\varvec{\theta }}$):

50 $$\begin{aligned} \Xi _m = \Xi _0 \cup \left\{ {\varvec{\mu }}_1 \right\} \cup \left\{ {\varvec{\mu }}_2 \right\} \cup \cdots \cup \left\{ {\varvec{\mu }}_m \right\} . \end{aligned}$$

The procedure stops at some step *M* (which can either be defined arbitrarily or by some tolerance on the value of the error indicator, and the value of the parameter that maximises the error indicator over the set $\Xi _M$ is then selected as $\mu _{{\text {max}}}^k$ (defined in Eq. (36)), where the exact solution will be computed:

51 $$\begin{aligned} \mu _{{\text {max}}}^k = \underset{\mu \in \Xi _m}{{\text {argmax}}}{\mathcal {J}}^m_\star (\mu ). \end{aligned}$$

**Fig. 14:**
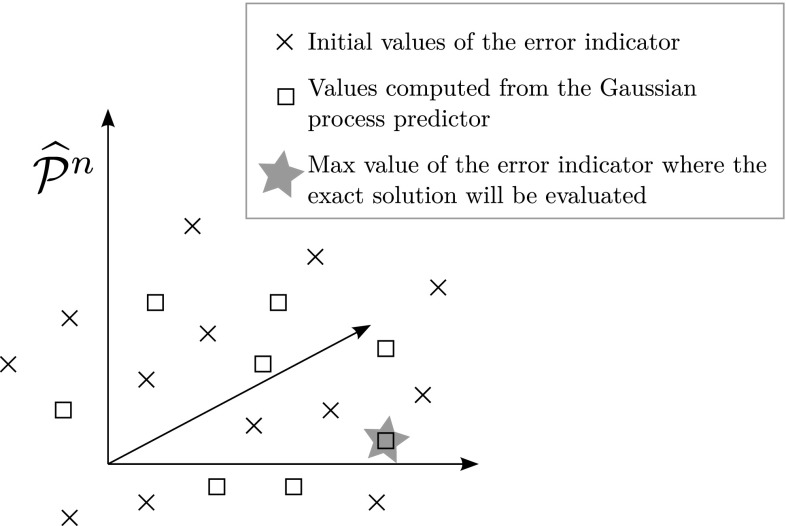
Computing the error indicator ${\mathcal {J}}$ for an initial arbitrary selection of parameters (denoted by the *crosses*), a Gaussian regression is iteratively computed to evaluate the indicator at locations where it is likely to be the highest (shown as the *squares*). Eventually, the parameter value selected is the one where the indicator indeed is the highest among this discrete set (shown as the *star*)

This is graphically sketched in Fig. 14. The process is also described in Algorithm 3.

**Fig. 15:**
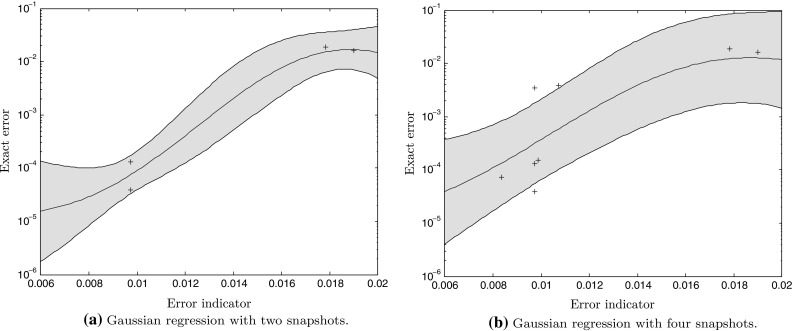
Evolution of the map between error indicator and exact error as the number of observations (snapshots) increases. From each snapshot, two errors can be computed: one before the enrichment of the basis and one afterwards. This allows to include more points to build the map


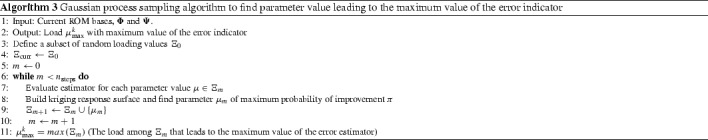


#### Construction of a Gaussian regression between the exact error and the error estimator to monitor the convergence of the procedure

**Fig. 16:**
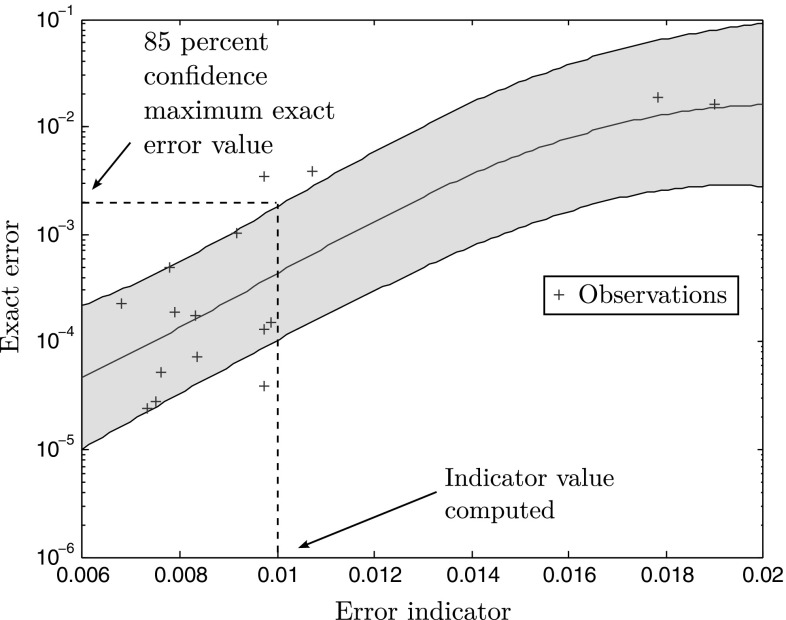
Gaussian process regression between error estimator and exact error, with $70\,\%$ confidence interval. Given the current observations (i.e., the snapshots in our reduced-order modelling jargon), a Gaussian regression is performed to establish a map between exact error and error estimator with statistical knowledge, which gives a confidence interval. Given a value of the error indicator, the user can use the map to have a safe estimate of the exact error using the confidence interval so as not to underestimate the error

One important matter in this procedure is that the error indicator (the norm of the residual in our case), for computational saving reasons, is driving the control of our algorithm. Beyond a certain proportionality, which drives the algorithm assuming smaller error indicators lead to smaller errors, there is no control on the magnitude of the actual error. This is a problem as we would like to build a reduced model that is accurate up to a certain tolerance that is chosen by the user. Hence, it is necessary to build some sort of map between estimator and error, which can then be used as a stopping criterion in our greedy algorithm by linking the value of the indicator to the actual error. This problem has been treated in [43] by using a linear regression using data samples obtained from the snapshots, that regression being updated each time a new snapshot is available. In this paper, we use again a Gaussian regression (in a similar way to [55]), just like it was done for the regression of the error indicator against the parameters. However, in this case, we consider noise, since there is not an exact monotonic match between error indicator and exact error a priori. This implies that the covariance expression changes slightly:

52 $$\begin{aligned} cov_n(\mathbf {x},\,\mathbf {y}) = \sigma ^2 \exp \left( \left( \mathbf {x^p} - \mathbf {x^q}\right) ^T\mathbf {I}_{\varvec{\theta }} \left( \mathbf {x^p} - \mathbf {x^q}\right) \right) + \sigma _n^2 \delta _{pq},\nonumber \\ \end{aligned}$$

where $\delta _{pq}$ is the Kronecker delta, and $\sigma _n^2$ is the variance of a noise assumed Gaussian, which is a new hyperparameter. Again, more details can be found in [53]. The main advantage over a linear regression is that it provides a more flexible fit as well as a confidence interval that can be used to ensure a bound on the error. Examples of regression with confidence intervals of one standard error from various numbers of data samples are displayed in Figs. 15 and 16. We define $\widehat{{\mathcal {Q}}^{+}},$ the “pessimistic” estimate of the quantity of interest ${\mathcal {Q}}^{{\text {HR}}},$ computed from a value of the error indicator ${\mathcal {J}}^{{\text {HR}}}$ through the Gaussian map ${\mathcal {G}}$ plus one standard deviation $\sigma$ (estimated from the data in the Gaussian process regression), which gives about an $85\,\%$ confidence that the actual error is below this value:

53 $$\begin{aligned} \widehat{{\mathcal {Q}}^{+}} = {\mathcal {G}}\left( {\mathcal {J}}^{{\text {HR}}}\right) + \sigma . \end{aligned}$$

#### Optimal choice of the size of the reduced spaces to achieve a user-defined tolerance

One important matter, once a new solution has been computed together with a singular value decomposition of its error on the current reduced basis ${\varvec{\Phi }},$ is to choose how many basis vectors $({{\varvec{\phi }}_{{\text {add}}}}_i)_{i = 1,\ldots ,n_{{\text {add}}}}$ should be concatenated to the basis ${\varvec{\Phi }},$ so that the ROM achieves some user-defined target tolerance $\epsilon .$ The same question goes for the number of basis vectors ${\varvec{\Psi }}$ representing the internal forces for the system approximation.

We choose to tackle this issue in two stages, by first making sure the size of the displacement reduced basis ${\varvec{\Phi }}$ is large enough for ${\mathcal {Q}}^{{\text {R}}}$ to achieve a certain fraction of the tolerance $\epsilon$, and then choosing the dimension of ${\varvec{\Psi }}$ to achieve the tolerance as well as insuring a monotonic decrease of the reduced and hyperreduced residuals ${\mathcal {J}}^{{\text {R}}}$ and ${\mathcal {J}}^{{\text {HR}}}.$

The procedure starts by computing an initial solution, chosen for an arbitrary value of the parameter $\mu ,$ as well as its residuals ${\mathcal {J}}^{{\text {R}}}_{ini},\,{\mathcal {J}}^{{\text {HR}}}_{ini}.$ These two residuals will be used as initial residual tolerances.

*Determining the size of the displacement reduced basis* ${\varvec{\Phi }}$ assume we are at step *k* of the greedy algorithm. We denote the displacement basis ${\varvec{\Phi }}^k.$ The snapshot was enriched with a new exact solution whose projection error with the current reduced basis ${\varvec{\Phi }}^k$ was decomposed into a POD expansion ${\varvec{\Phi }}_{{\text {add}}}$, i.e., $e_{{\text {proj}}} \simeq \sum _i^{n_{{\text {add}}}} \alpha _i {{\varvec{\phi }}_{{\text {add}}}}_i.$

We successively evaluate the quantity of interest ${\mathcal {Q}}^{{\text {R}}}$ (defined in Eq. (29)) with an increasing number of basis vectors until ${\mathcal {Q}}^{{\text {R}}} < {\gamma ^{{\text {R}}}_Q}\cdot \epsilon$ and ${\mathcal {J}}^{{\text {R}}} < \nu ^{{\text {R}}}_{{\text {current}}},$ where $\gamma ^{{\text {R}}}_Q$ is a scalar smaller than 1 that forces the reduced model (not hyperreduced) to achieve “comfortably” the tolerance $\epsilon ,$ allowing the hyperreduced model, which is an approximation of the reduced model, to actually achieve the tolerance $\epsilon .$ In mathematical terms, this can be written:

54 $$\begin{aligned}&\underset{}{{\text {min}}} \,{\text {dim}}\left( {\varvec{\Phi }}_{{\text {add}}}\right) \,\textit{such \, that} \, {\mathcal {Q}}^{{\text {R}}}\left( {\varvec{\Phi }}^{k+1}\right) < {\gamma ^{{\text {R}}}_Q}\cdot \epsilon \nonumber \\&\quad \text {and}\quad {\mathcal {J}}^{{\text {R}}}\left( {\varvec{\Phi }}^{k+1}\right) < \nu ^{{\text {R}}}_{{\text {current}}}, \end{aligned}$$

where ${\varvec{\Phi }}^{k+1} = [{\varvec{\Phi }}^{k},\, {\varvec{\Phi }}_{{\text {add}}}]$ (i.e., ${\varvec{\Phi }}^{k+1}$ is the concatenation of ${\varvec{\Phi }}^{k}$ and ${\varvec{\Phi }}_{{\text {add}}}$).

The residual tolerance is updated as: $\nu ^{{\text {R}}}_{{\text {current}}} = {\gamma ^{{\text {R}}}_\nu }\cdot \nu ^{{\text {R}}}_{{\text {current}}},$ with ${\gamma ^{{\text {R}}}_\nu }<1.$ The condition on the residual ensures its decrease throughout the procedure. This is important since the indicator quantity ${\mathcal {J}}^{{\text {R}}}$ (influencing ${\mathcal {J}}^{{\text {HR}}}$) drives the procedure, the exact error being used for the stopping criterion only (through the Gaussian process regression between error indicator and actual error). The value of $\nu ^{{\text {R}}}_{{\text {current}}}$ is initialized with the value of the initial residual of the initial ROM, whose size is chosen minimal, typically only of dimension 1 to start with.

Note that this step is quite expensive, as it requires to evaluate the reduced solution several times with no hyperreduction. It could be made cheaper by substituting the evaluations of the non-hyperreduced ROM by a finely (i.e., with a high-dimensional basis ${\varvec{\Psi }}$) hyperreduced ROM which would be cheaper to evaluate. However, the construction of the hyperreduction ROM is expensive in itself since it requires evaluation of the non-hyperreduced counterpart to build the snapshot necessary to build the internal forces basis ${\varvec{\Psi }}.$ A trade-off would have to be found. In our case, we keep the strategy as it is, keeping in mind that although computationally intensive, this procedure is performed offline.

*Determining the size of the internal forces reduced basis* ${\varvec{\Psi }}$ in a similar way, we will successively evaluate the quantity of interest ${\mathcal {Q}}^{{\text {HR}}}$ with an increasing number of basis vectors until the tolerance is reached for all the values of the parameter in the current snapshot:

55 $$\begin{aligned}&\underset{}{{\text {min}}} \,{\text {dim}}({\varvec{\Psi }})\,\textit{such \, that}\,\left( \max _{\mu \in \Xi _k}{{\mathcal {Q}}^{{\text {HR}}}({\varvec{\Psi }},\,\mu )}\right) < \epsilon \nonumber \\&\quad {\text {and}}\quad \left( \max _{\mu \in \Xi _k}{\mathcal {J}}^{{\text {HR}}}({\varvec{\Psi }})\right) < \nu ^{{\text {HR}}}_{{\text {current}}}. \end{aligned}$$

In this case, the tolerance $\epsilon$ has to be reached for all solutions in the current snapshot $\Xi _k,$ and not only the last one computed. This guarantees the stability of the method. Indeed, unlike when enriching the displacement basis ${\varvec{\Phi }},$ there is no guarantee on the monotonic decrease of the quantity of interest. To guarantee the monotonic convergence of the error indicator, the residual is updated at each step: $\nu ^{{\text {HR}}}_{{\text {current}}} = {\gamma ^{{\text {HR}}}_\nu }\nu ^{{\text {HR}}}_{{\text {current}}}.$ The general construction of the reduced basis within one pseudo-parameter space $\widehat{{\mathcal {P}}}^n$ is described in Algorithm 4.

##### *Remark*

Note that this step is not computationally expensive since it only requires evaluations of the hyperreduced model.


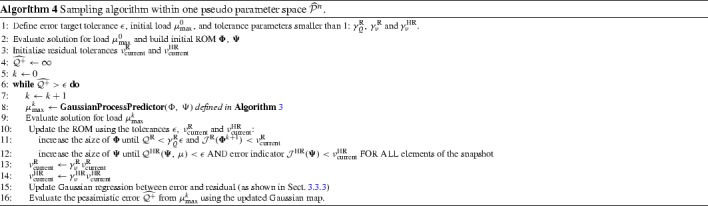

**Fig. 17:**
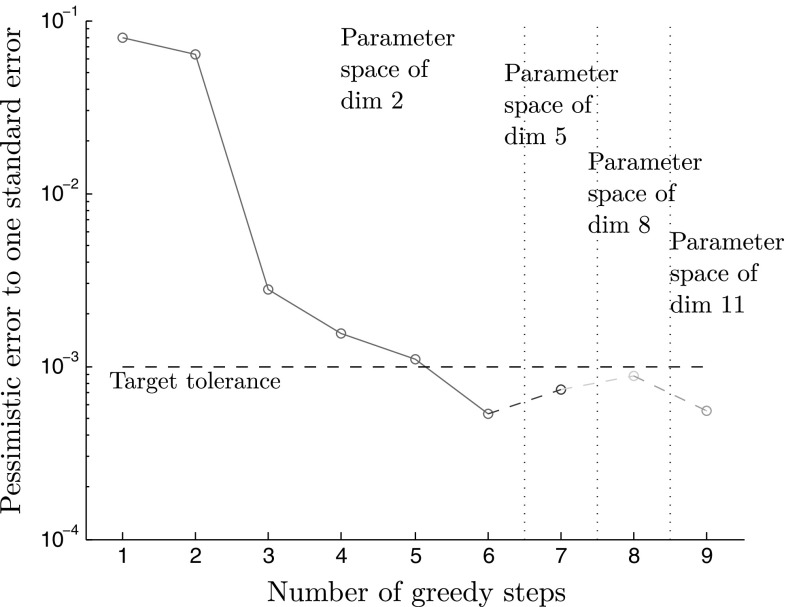
Evolution of the pessimistic error $\widehat{{\mathcal {Q}}^{+}}$ (defined in Eq.  (53)) inside and across pseudo-parameter spaces. The error is computed from the error indicator using the Gaussian process predictor plus one standard error as explained in Sect. 3.3.2. The ROM is first build based on solutions from pseudo parameter space $\widehat{{\mathcal {P}}}^0$ that is of dimension 2 up to reaching the tolerance $\epsilon = 10^{-3}.$ Once the tolerance is reached, pseudo parameter space $\widehat{{\mathcal {P}}}^1$ of dimension 5 is considered. Because the first evaluation of the error is already achieving the tolerance $\epsilon ,$ the procedure moves on to space $\widehat{{\mathcal {P}}}^2$ of dimension 8 which also achieves this tolerance straight away. Moving onto the space $\widehat{{\mathcal {P}}}^3$ of dimension 11, the error decreases: one can consider that convergence has been achieved for parameter spaces of any size

#### Application of the Bayesian POD-greedy algorithm

We now proceed to apply the POD-greedy Algorithms 2–4 on the RVE problem described in Sect.  2. We define the target tolerance $\epsilon = 10^{-3},\,\gamma ^{{\text {R}}}_Q = \frac{1}{2},\,\gamma ^{{\text {R}}}_\nu = \frac{1}{2}$ and $\gamma ^{{\text {HR}}}_\nu = 0.9.$ We proceed to build a ROM achieving tolerance $\epsilon$ on the successive pseudo parameter spaces $\widehat{{\mathcal {P}}}$ of dimensions 2, 5 and 8. The very initial parameter value is the proportional loading of equal value in all directions (that is in $\epsilon _{xx},\,\epsilon _{yy}$ and $\epsilon _{xy}$). Results are displayed in Fig. 17.

After achieving the tolerance for snapshots in the initial pseudo parameter space of dimension 2, the pessimistic value of the error (up to one standard error) $\widehat{{\mathcal {Q}}^{+}}$ increases slightly when moving on to the space of dimension 5. This is not surprising since the ROM was constructed to achieve the tolerance on the space of dimension 2 and does not represent as well the space of dimension 5. However, this error increase is small and remains underneath the target tolerance $\epsilon .$ Moving on to the space of dimension 8 leads to the same analysis. When considering the space of dimension 11, we can see that the error decreases. This means that despite the last computed solution belongs to a space of larger dimension (and is the least well represented one) than the space used to build the ROM, it is correctly approximated. One can then argue that the current ROM is accurate enough to represent the solutions issued from parameter spaces of any dimension. Hence, there is no need to consider any finer spaces and the procedure can stop there.

##### *Remark*

For dimension 8 and 11, we used a quasi-random Latin-hypercube sampling [56] rather that the Gaussian process regression described in Sect. 3.3.3. Indeed, in higher dimensions, the Gaussian process regression requires proportionally more data to make sensible predictions.

## Conclusion and perspectives

In this paper, we have developed an offline/online reduced basis strategy to reduce the computational cost of solving the RVE boundary value problem involved in nested computational homogenisation (i.e., $\mathrm{FE}^2$). Our strategy has been illustrated in the context of elastic-damageable particulate composites. Such problems can be parametrised by the history of the far-fields that are applied as boundary conditions to the RVE. The main challenge comes from the very high-dimensionality of the parameter domain which consists of all possible macroscopic load histories that preserve material stability. This makes the sampling of the parameter space, which is necessary to train projection-based reduced models, a complicated task. We have proposed two strategies to solve this problem:

- The sampling is done randomly, in a brute force manner, whilst enforcing that a certain increment of energy dissipation occurs at each timstep of the discrete load history. The reduced space is found by using the POD.
- The problem is solved using a POD-greedy reduced-basis method. To reduce the dimensionality of the RVE problem to tractable levels, the parameter space is substituted by a hierarchy of approximate spaces of small and increasing dimensions. A ROM is computed for each of these approximate spaces, using a POD-greedy training algorithm, in conjunction with a Bayesian-optimisation-based a posteriori error estimate.

The brute force strategy can be very expensive when requiring a high accuracy of the ROM. Indeed, despite the minimum dissipation constraint, the exhaustiveness of the snapshot requires a large number of random solutions. This approach can be appealing nevertheless if the accuracy required is relatively low, due to its low computational cost and ease of implementation. On the other hand, the hierarchical reduced basis strategy is exploring the parameter space in a robust and quasi-optimal manner, at the cost of a certain algorithmic complexity.

Coming back to the context of multiscale modelling, we can question the necessity of computing reduced models of RVE problems in the space of arbitrary far-field loads. Indeed, in practical applications, only specific loadings may actually be applied to the RVE, making the pursuit of an exhaustive snapshot irrelevant. We are currently investigating the possibility of integrating some knowledge about potential macroscopic solutions in order to restrict the size of the parameter domain a priori.

## Footnotes

1 We make the formulation in a 2D context, but the same principles apply in 3D.

2 It is not 3 since we fixed the value of the load between two successive time steps.

3 Some authors advocate the need for a residual minimising approach to ensure the optimality of this step [32, 41].

4 This is arguable as the gradient-based optimizer will converge to a local minimum in the parameter domain, see [39] for a more detailed discussion and the proposition of a remedy.

5 We work at a fully discrete level with vectors of degrees of freedom corresponding to continuous fields that belong to FE spaces, but we will refer to such quantities as “fields” or simply “displacements” to avoid unnecessary complication of the explanations.

6 Note that in practice, $n_t$ is different between different load cases. Here we try to keep simple notations.

7 To simplify the notations we denote ${\mathbf {f}}_{{\text {int}}}({\bar{\mathbf {u}}} + {\varvec{\Phi }} {\varvec{\alpha }})$ by simply ${\mathbf {f}}_{{\text {int}}}({\varvec{\Phi }} {\varvec{\alpha }})$ in the remaining of this paper.
